# Nitrogen Homeostasis Reveals an Internal Inconsistency in the Energy Balance Model’s Predicted Coincidence of Energy Balance and Body Weight Stability

**DOI:** 10.3390/biology15201817

**Published:** 2026-10-12

**Authors:** Francisco Arencibia-Albite

**Affiliations:** School of Health and Sciences, Universidad del Sagrado Corazón, P.O. Box 12383, San Juan PR 00914-0383, Puerto Rico; franciscom.arencibia@sagrado.edu

**Keywords:** energy balance model, mass balance, nitrogen balance, body weight regulation, macronutrient fluxes

## Abstract

The Energy Balance Model predicts that energy balance coincides with body weight stability. The present analysis shows that this relationship emerges from the canonical equations of the model rather than from an assumption introduced by the study. Further analytical examination demonstrates that this predicted state corresponds to fat mass and fat-free mass stability and consequently requires nitrogen homeostasis. These conditions lead to a coupled mass- and energy-accounting system. When solved without enforcing nitrogen homeostasis, the equilibrium solution requires persistent carbohydrate accumulation, protein depletion, and negative nitrogen balance, a combination physiologically incompatible with a prolonged steady body weight. Conversely, enforcing the already-derived requirement of zero nitrogen balance eliminates these macronutrient imbalances but requires obligatory non-urinary nitrogen losses to vanish, which is physiologically impossible. Thus, the contradiction arises from the model’s equilibrium structure. By formal analytical reasoning, prolonged body weight stability must therefore coexist with energy imbalance, a conclusion consistent with observations of stable body weight despite apparent energy imbalance and randomized metabolic-ward data in which energy imbalance failed to correctly predict both the direction and magnitude of body weight change. Energy imbalance alone cannot sufficiently describe body weight dynamics because energetic quantities do not uniquely specify material fluxes.

## Simple Summary

Body weight is assumed to remain stable when the energy obtained from food equals the energy used by the body, a state known as energy balance. This study asks whether that relationship remains physiologically consistent when the material composition of the body is considered. Starting directly from the equations of the Energy Balance Model, the analysis shows that energy balance implies stable body weight and, in turn, stability of fat mass and fat-free mass. These conditions require long-term nitrogen balance because nitrogen-containing body material cannot continuously accumulate or disappear while body composition remains stable. However, when unavoidable non-urinary nitrogen losses are explicitly accounted for, the model cannot satisfy these requirements simultaneously. The contradiction arises from equilibrium conditions implicit in the model itself rather than from an additional assumption introduced in this study. Consequently, the analysis indicates that prolonged body weight stability must coexist with energy imbalance, a conclusion consistent with observations of stable body weight despite apparent energy imbalance and with metabolic-ward data in which measured energy imbalance failed to correctly predict both the direction and magnitude of body weight change. A possible reason is that the same amount of metabolic energy can correspond to different amounts of material entering, being oxidized, or physically leaving the body.

## 1. Introduction

Quantitative implementations of the Energy Balance Model (EBM) implicitly show that long-term body weight stability and energy balance (i.e., energy intake equals energy expenditure) coincide as equilibrium conditions [1,2,3,4,5,6,7,8,9,10,11,12,13,14,15,16,17]. This relationship emerges when the models approach their steady state, at which energy balance occurs simultaneously with the absence of body weight change. Energy balance and mass balance nevertheless represent distinct equilibrium constraints in open systems because they arise from different conservation principles [18,19]. This distinction raises a fundamental question: is the coincidence of energy balance and body weight stability predicted by the EBM compatible with the distinct material constraints necessarily associated with body mass equilibrium in an open biological system? If not, the analytical structure of the EBM may contain internal inconsistencies that become apparent when the macronutrient fluxes contributing to energy expenditure are represented explicitly. An important specific case of these material constraints concerns nitrogen homeostasis: prolonged body weight stability implicitly requires, on average, no sustained accumulation or depletion of nitrogen-containing body material and therefore a mean nitrogen balance equal to zero [20,21,22,23]. Accordingly, an internally consistent equilibrium should simultaneously satisfy energy balance, body mass equilibrium, and nitrogen homeostasis.

Previous analyses by Arencibia-Albite [24,25] demonstrated mathematically that the EBM-predicted coincidence of energy balance and body weight stability gives rise to internal inconsistencies. Those analyses demonstrated the inconsistencies by formulating and solving a coupled mass- and energy-accounting system without first deriving from the canonical EBM equations the equilibrium constraints that naturally lead to its formulation. As part of this work, Arencibia-Albite [24] also analyzed randomized controlled trial data collected under tightly supervised inpatient conditions and showed that food-mass intake exhibited a more systematic correspondence with observed body weight and body composition changes, whereas measured energy imbalance frequently failed to predict the direction and magnitude of body weight change. These empirical findings provided an independent line of evidence supporting the physiological relevance of distinguishing material accounting from energetic accounting in the analysis of body weight dynamics.

The present study extends these previous analyses by addressing a question that was not explicitly examined in references [24,25]: whether the equilibrium conditions derived directly from the canonical EBM equations necessarily imply nitrogen homeostasis as a physiological requirement of prolonged body weight stability and whether the resulting coupled mass- and energy-accounting system can satisfy this requirement. In particular, the analysis demonstrates that the coincidence of energy balance and body weight stability follows directly from the relationship between energy imbalance and changes in body composition, establishes that this equilibrium also requires stability of both fat mass and fat-free mass, and derives the corresponding condition that absorbed fat intake equals net fat oxidation. Because fat mass and fat-free mass together constitute total body mass, their prolonged stability further implies that a mean nitrogen balance equal to zero is an implicit physiological requirement of the equilibrium predicted by the canonical EBM equations. Collectively, these analytical developments make explicit the need for a coupled mass- and energy-accounting system. Thus, whereas the coupled accounting system itself was introduced previously [24,25], the principal novelty of the present study is the derivation of the constraints leading to its formulation directly from the canonical EBM framework, including the demonstration of body composition stability and nitrogen homeostasis as necessary consequences of the prolonged equilibrium predicted by that framework, followed by evaluation of whether the resulting equilibrium can satisfy these requirements.

Building upon this analytical foundation, this manuscript further examines the implications of the identified inconsistency for body weight regulation. Specifically, it considers the logical consequence of the EBM-derived contradiction for the relationship between energy balance and prolonged body weight stability and evaluates whether energy imbalance alone can serve as a sufficient descriptor of body weight dynamics. The analysis then examines the relationship between energetic quantities and their underlying material fluxes using substrate-oxidation and indirect-calorimetry equations, demonstrating that the same energy expenditure can correspond to different amounts of oxidized substrate and metabolically associated material elimination. This non-unique correspondence may explain why metabolic energy and material fluxes should be treated as distinct descriptions, since body mass change is determined directly by the balance of material entering and leaving the body. Because the inconsistency emerges directly from the canonical EBM equations, the analytical conclusion does not depend on any particular experimental dataset. Previously reported empirical findings therefore serve only as complementary evidence.

## 2. Methods

### 2.1. Definition of Prolonged Body Weight Stability

In the present analysis, a distinction is made between exact mathematical steady-state and the approximate body weight stability observed physiologically. Mathematical equilibrium represents an idealized steady-state in which body weight (*BW*) exhibits no change and is therefore expressed as *dBW*/*dt* = 0. Physiological body weight, by contrast, may undergo short-term fluctuations because of variations in body water, gastrointestinal contents, glycogen stores, and other transient factors while remaining approximately stable around a long-term mean. Such fluctuations do not invalidate a steady-state representation when the analysis concerns the long-term behavior of the system. Accordingly, prolonged body weight stability is defined here as a state in which body weight exhibits no sustained increase or decrease over the relevant long-term interval. Throughout the analytical development, the notation *dBW*/*dt* = 0 is used to represent this long-term steady state condition rather than the literal absence of short-term fluctuations in measured body weight.

### 2.2. Study Organization

The present study evaluates the internal consistency of the EBM by analytically examining the equilibrium consequences that follow from its governing equations. The analysis begins by presenting the equations commonly used in quantitative formulations of the EBM, including the fundamental relationship between energy imbalance and changes in body composition and the corresponding equation describing fat mass dynamics.

The analytical consequences of these equations are then developed sequentially. First, it is demonstrated that, within the EBM framework, energy balance (*EI* = *EE*) necessarily implies body weight stability (*dBW*/*dt* = 0). Because body weight represents body mass, conservation of mass further requires that this EBM-predicted equilibrium correspond to equality between total material inflow and outflow. The analysis then shows that the same equilibrium requires stability of both fat mass and fat-free mass and therefore precludes sustained accumulation or depletion of nitrogen-containing body material. Consequently, a mean nitrogen balance equal to zero emerges as an implicit physiological requirement of the prolonged body weight equilibrium predicted by the EBM rather than as an additional equilibrium assumption imposed in the present work.

These equilibrium requirements are subsequently expressed quantitatively. The relationship between absorbed fat intake and net fat oxidation is derived, nitrogen balance is formulated in terms of protein intake, protein oxidation, and obligatory non-urinary nitrogen losses, and the mass associated with these heterogeneous nitrogen-containing losses is distinguished from their nitrogen content. These relationships are then incorporated into a coupled mass- and energy-accounting system representing the simultaneous conditions of energy balance and body weight stability predicted by the EBM. The system is first solved without explicitly imposing nitrogen homeostasis and then solved again subject to the previously derived requirement that nitrogen balance equal zero. Comparison of the two solutions provides the basis for determining whether the EBM-predicted prolonged equilibrium is physiologically self-consistent once obligatory non-urinary nitrogen losses are explicitly incorporated into the material accounting. Finally, the implications of the identified inconsistency for the interpretation of body weight regulation are examined. The analysis considers the distinction between energetic quantities and their underlying material fluxes and uses indirect calorimetry equations to illustrate that the same energy expenditure can correspond to different amounts of oxidized substrate and metabolically associated material elimination under physiologically plausible conditions. These results are used to evaluate whether energy imbalance alone can uniquely specify the material imbalance governing changes in body mass. Detailed mathematical derivations, including the stoichiometric development of the indirect calorimetry equations and additional analytical steps omitted from the main text, are provided in the Appendix A.

## 3. Results

### 3.1. Canonical Equations of the EBM

In the EBM framework, total body energy content changes according to the difference between daily energy intake, *EI* (kcal/day), and daily energy expenditure, *EE* (kcal/day) [26]. Expressed quantitatively, this relationship takes the following form:

(1) $$\mathrm{EI}\;-\;\mathrm{EE}=\underset{\begin{array}{c} \mathrm{Rate}\;\mathrm{of}\;\mathrm{Change}\;\mathrm{in}\;\\ \mathrm{Total}\;\;\mathrm{Body}\;\mathrm{Energy}\; \end{array}}{\underset{︸}{\overset{\begin{array}{c} \mathrm{Rate}\;\mathrm{of}\;\mathrm{Change}\;\mathrm{in}\\ \mathrm{Fat}\;\mathrm{Mass}\;\mathrm{Energy}\; \end{array}}{\overset{︷}{\rho_{\mathrm{FM}}\frac{\mathrm{dFM}}{\mathrm{dt}}}}\;\;+\;\;\overset{\begin{array}{c} \mathrm{Rate}\;\mathrm{of}\;\mathrm{Change}\;\mathrm{in}\\ \mathrm{Fat}-\mathrm{Free}\;\mathrm{Mass}\;\mathrm{Energy}\; \end{array}}{\overset{︷}{\rho_{\mathrm{FFM}}\frac{\mathrm{dFFM}}{\mathrm{dt}}}}}}$$

where *FM* and *FFM* represent fat mass and fat-free mass, respectively; *ρ_X_* is the energy density (kcal/kg) associated with body compartment *X*; and *t* denotes time in days [13,27,28]. This equation has been fundamental for calculating energy imbalance as a function of diet-induced changes in body composition [27,28].

Because changes in body composition are central to quantitative implementations of the EBM, explicit accounting relationships for fat mass dynamics are also required. In particular, the temporal evolution of fat mass is governed by the following expression: the daily rate of change in fat mass, *dFM*/*dt*, equals the difference between the daily absorbed fat intake (*FI_a_*, g/day) and the daily net of fat oxidation (*FOx_net_*, g/day) [29]:

(2) $$\frac{\mathrm{dFM}}{\mathrm{dt}}=FI_{a}-\mathrm{FO}x_{\mathrm{net}}$$

Net fat oxidation is determined by subtracting the rate of de novo lipogenesis from total fat oxidation, since newly synthesized lipid offsets part of the fat mass lost through oxidation [29,30]. Equation (2) has played a central role in investigations of how diet composition influences adiposity by linking dietary fat intake and net fat oxidation to changes in fat mass [29].

Detailed derivations of Equations (1) and (2) are provided in Appendices A.1 and A.2.

### 3.2. Energy Balance and Body Weight Stability Coincide Within the EBM Framework, Implying Simultaneous Energy, Mass, and Nitrogen Equilibrium

Implicit in Equation (1) is the central EBM relationship between energy imbalance and the rate of body weight change. This relationship is demonstrated below.

The rate of body weight change is the sum of rates of change in fat mass and fat-free mass:

$$\frac{\mathrm{dBW}}{\mathrm{dt}}=\frac{\mathrm{dFM}}{\mathrm{dt}}\;\;+\;\;\frac{\mathrm{dFFM}}{\mathrm{dt}}$$

This equation can be rearranged into two alternative forms (see Appendix A.3):

$$\begin{array}{c} \frac{\mathrm{dFM}}{\mathrm{dt}}=\left[\frac{1}{1\;\;+\;\;\mathrm{dFFM}/\mathrm{dFM}}\right]\frac{\mathrm{dBW}}{\mathrm{dt}},\\ \frac{\mathrm{dFFM}}{\mathrm{dt}}=\left[\frac{\mathrm{dFFM}/\mathrm{dFM}}{1\;\;+\;\;\mathrm{dFFM}/\mathrm{dFM}}\right]\frac{\mathrm{dBW}}{\mathrm{dt}} \end{array}$$

Substituting both expressions into Equation (1) and solving for *dBW*/*dt* yields:

(3) $$\frac{\mathrm{dBW}}{\mathrm{dt}}=\frac{\mathrm{EI}-\mathrm{EE}}{\rho_{\mathrm{BM}}}$$

where *ρ_BM_* > 0 denotes the energy density of body mass, which varies with body composition [31,32] and is defined as follows:

$$\rho_{\mathrm{BM}}=\frac{\rho_{\mathrm{FM}}\;\;+\;\;\rho_{\mathrm{FFM}}\mathrm{dFFM}/\mathrm{dFM}}{1\;\;+\;\;\mathrm{dFFM}/\mathrm{dFM}}$$

A detailed derivation of Equation (3) is provided in Appendix A.4.

The previous demonstration shows that Equation (3) is mathematically equivalent to Equation (1) while explicitly expressing the rate of body weight change as the ratio of energy imbalance to body mass energy density. Moreover, Equation (3) makes the EBM condition for body weight stability (*dBW*/*dt* = 0) explicit: if energy intake equals energy expenditure (*EI* = *EE*), then body weight must remain stable, that is:

$$\mathrm{EI}=\mathrm{EE}\;\;\;\;\Rightarrow \;\;\;\;\frac{\mathrm{dBW}}{\mathrm{dt}}=\frac{\mathrm{EI}-\mathrm{EE}}{\rho_{\mathrm{BM}}}=0$$

Consequently, within the analytical framework of the EBM, energy balance (*EI* = *EE*) coincides with body weight stability, even though this relationship is not immediately apparent from qualitative inspection of Equation (1).

Additionally, because body weight represents body mass, this condition also has an obligatory material consequence. By conservation of mass,

$$\frac{\mathrm{dBW}}{\mathrm{dt}}=M_{\mathrm{inflow}}-\;M_{\mathrm{outflow}}$$

so that

$$\frac{\mathrm{dBW}}{\mathrm{dt}}=0\;\;\;\;\Rightarrow \;\;\;\;\;M_{\mathrm{inflow}}\;=M_{\mathrm{outflow}}$$

where *M*_inflow_ and *M*_outflow_ denote the rates of mass inflow and mass outflow, respectively (kg/day). Thus, the coincidence of energy balance and body weight stability implicitly predicted by the EBM necessarily corresponds to the simultaneous occurrence of energy and mass equilibrium: energy entering the body must equal, on average, energy leaving it, while material entering the body must equal, on average, material leaving it. Importantly, the latter condition is neither an additional equilibrium assumption nor an interpretation-dependent feature of the present analysis. Rather, it is a hidden condition of the EBM framework that, as demonstrated by the preceding analytical steps, naturally emerges from Equation (1), the fundamental EBM equation relating energy imbalance to changes in total body energy.

At a prolonged physiological steady-state, this whole-body mass equilibrium further requires that no body constituent undergo sustained accumulation or depletion. Accordingly, the material fluxes determining the amount of each body constituent must collectively yield no sustained net accumulation or depletion over the relevant time interval. This requirement is particularly important for nitrogen: a persistent positive or negative nitrogen balance would imply progressive accumulation or depletion, respectively, of nitrogen-containing body material and therefore would be incompatible with a true long-term body mass steady-state. Just as body weight stability does not appear explicitly in the fundamental EBM equation (Equation (1)) but emerges from its analytical development, a mean nitrogen balance equal to zero also emerges as a necessary condition of the resulting prolonged body weight equilibrium, even though nitrogen balance is not explicitly represented in the canonical EBM equations. Therefore, the fact that a condition is not explicitly represented in Equation (1) does not imply that it has been externally imposed on the EBM when that condition follows necessarily from the equilibrium state predicted by the framework. This implication is further supported analytically in the following section through the compartment-level relationships among body weight, fat mass, and fat-free mass that follow from the EBM equations.

Finally, because absorbed carbohydrate, fat, and protein contribute to both energy and mass inflow, while their metabolic processing contributes to both energy expenditure and mass outflow, the same macronutrient fluxes must satisfy both accounting requirements simultaneously. Determining the relationships among these intake and oxidation fluxes therefore requires a coupled mass- and energy-accounting system. Before formulating this system explicitly, however, two relationships must first be developed quantitatively: the relationship between absorbed fat intake and net fat oxidation under these equilibrium conditions, and a formal nitrogen balance expression that makes explicit how nitrogen homeostasis enters the mass- and energy-accounting relationships.

### 3.3. Within the EBM Framework, the Coincidence of Energy Balance and Body Weight Stability Implies Fat Mass and Fat-Free-Mass Equilibrium

An analytical consequence of the EBM framework is that, at the coincidence of energy balance and body weight stability, absorbed fat intake (*FI_a_*) must equal net fat oxidation (*FOx_net_*), as demonstrated next.

The rate of change in fat-free mass must equal the rate of change in the difference between body weight and fat mass:

$$\frac{\mathrm{dFFM}}{\mathrm{dt}}=\frac{d}{\mathrm{dt}}\left(\mathrm{BW}-\mathrm{FM}\right)=\frac{\mathrm{dBW}}{\mathrm{dt}}\;\;-\;\;\frac{\mathrm{dFM}}{\mathrm{dt}}$$

Substituting this expression into Equation (1) yields:

$$\mathrm{EI}\;-\;\mathrm{EE}=\left(\rho_{\mathrm{FM}}-\rho_{\mathrm{FFM}}\right)\frac{\mathrm{dFM}}{\mathrm{dt}}\;+\;\rho_{\mathrm{FFM}}\frac{\mathrm{dBW}}{\mathrm{dt}}$$

Substituting Equation (2) gives:

$$\mathrm{EI}\;-\;\mathrm{EE}=\left(\rho_{\mathrm{FM}}-\rho_{\mathrm{FFM}}\right)\left(FI_{a}-\mathrm{FO}x_{\mathrm{net}}\right)\;\;+\;\rho_{\mathrm{FFM}}\frac{\mathrm{dBW}}{\mathrm{dt}}$$

Thus, when energy balance (*EI* − *EE* = 0) and body weight stability (*dBW*/*dt* = 0) coincide, the preceding expression reduces to:

(*ρ_FM_* − *ρ_FFM_*)(*FI_a_* − *FOx_net_*)   = 0

Consequently, since *ρ_FM_ − ρ_FFM_* ≠ 0 [27], the only possible solution is:

*FI_a_* = *FOx_net_*

which completes the demonstration and shows that, at the coincidence of energy balance and body weight stability, fat mass is also stable:

$$\frac{\mathrm{dFM}}{\mathrm{dt}}=FI_{a}\;-\mathrm{FO}x_{\mathrm{net}}=0$$

This conclusion has an additional implication for the equilibrium state predicted by the EBM. As demonstrated above, the coincidence of energy balance (*EI* = *EE*) and body weight stability (*dBW*/*dt* = 0) yields a stable fat mass (*dFM*/*dt* = 0). Substitution of these conditions into the expression for the rate of change in fat-free mass therefore gives:

$$\frac{\mathrm{dFFM}}{\mathrm{dt}}=\frac{\mathrm{dBW}}{\mathrm{dt}}\;\;-\;\;\frac{\mathrm{dFM}}{\mathrm{dt}}=0$$

Consequently, because both fat mass and fat-free mass are stable at this equilibrium, neither compartment can undergo the sustained accumulation or depletion of nitrogen-containing body material that would result from a persistent positive or negative nitrogen balance. Such a persistent nitrogen imbalance is therefore incompatible with the simultaneous conditions *dFM*/*dt* = 0 and *dFFM*/*dt* = 0. Thus, the result derived above provides a direct compartment-level demonstration that the prolonged equilibrium predicted by the EBM implicitly requires a mean nitrogen balance equal to zero. The analytical sequence leading from the canonical EBM equation (Equation (1)) to the simultaneous steady-state conditions of body weight, fat mass, fat-free mass, and nitrogen balance is summarized in Figure 1.

### 3.4. Quantitative Formulation of the Nitrogen Balance Requirement of Prolonged Body Weight Stability

As analytically established in Section 3.3, a mean nitrogen balance equal to zero is an implicit physiological requirement of the prolonged body weight equilibrium predicted by the EBM (Figure 1). To formulate this requirement quantitatively, daily nitrogen balance (*NB*, in g/day) can be expressed as follows:

*NB* = *N*_in_ − *N*_urine_ − *N*_insensible_ (4)

where *N*_in_ represents dietary nitrogen intake, *N*_urine_ represents urinary nitrogen excretion, and *N*_insensible_ represents obligatory non-urinary nitrogen losses through feces, sweat, skin, and other minor routes.

The compartment-level basis of this requirement is that, at the coincidence of energy balance and body weight stability, both fat mass and fat-free mass are stable (*dFM*/*dt* = 0 and *dFFM*/*dt* = 0), as demonstrated in Section 3.3. Because these compartments together constitute total body mass within the EBM framework, a persistent nonzero nitrogen balance cannot coexist indefinitely with this equilibrium. A sustained positive nitrogen balance would imply progressive accumulation of nitrogen-containing body material, whereas a sustained negative nitrogen balance would imply progressive depletion. Changes in body compartments could temporarily accommodate such nitrogen imbalances over finite observation intervals, allowing for approximately stable total body weight despite a nonzero nitrogen balance. Such changes, however, cannot occur persistently at the prolonged equilibrium considered here, for which both *dFM*/*dt* = 0 and *dFFM*/*dt* = 0. Consequently, the long-term mean nitrogen balance must satisfy *NB* = 0.

To express nitrogen balance in terms of the protein fluxes used in the energy-accounting equations, let *k_P_* denote the conventional nitrogen-to-protein-equivalent conversion factor [29,30,33,34]. Thus,

$$N_{\mathrm{in}}=\frac{PI_{a}}{k_{P}}$$

and

$$N_{\mathrm{urine}}=\frac{\mathrm{PO}x_{\mathrm{net}}}{k_{P}}$$

where *PI_a_* denotes absorbed protein intake and *POx_net_* denotes the corresponding net protein-oxidation, which represents irreversible amino acid catabolism and associated gluconeogenic losses. Substitution into Equation (4) gives:

(5) $$\mathrm{NB}=\frac{PI_{a}-\mathrm{PO}x_{\mathrm{net}}}{k_{P}}\;\;-\;\;\;N_{\mathrm{insensible}}$$

Therefore, enforcing the EBM-implied requirement of nitrogen homeostasis, *NB* = 0, yields:

(6) $$N_{\mathrm{insensible}}=\frac{PI_{a}-\mathrm{PO}x_{\mathrm{net}}}{k_{P}},$$

and equivalently,

*PI_a_* = *POx_net_* +   *k_P_N*_insensible_. (7)

Because obligatory non-urinary nitrogen losses are positive,

*N*_insensible_ > 0, (8)

and *k_P_* > 0, Equation (7) requires:

*PI_a_* > *POx_net_* (9)

Thus, at nitrogen homeostasis, absorbed protein intake must compensate not only for the protein-equivalent associated with irreversible amino acid catabolism but also for nitrogen eliminated through obligatory non-urinary routes.

An additional distinction is required between the nitrogen flux represented by *N*_insensible_ and the mass of the heterogeneous nitrogen-containing material through which this nitrogen is eliminated. The conventional factor *k_P_* appearing in Equations (5)–(7) converts nitrogen into a protein equivalent and should not be assumed to represent the exact material-mass-to-nitrogen-mass ratio of all non-urinary nitrogen-containing losses. If *N*_j_ denotes the nitrogen mass associated with the *j*th class of eliminated material and *k*_j_ denotes its corresponding material-to-nitrogen mass ratio, then the total mass of non-urinary nitrogen-containing material eliminated (*M*_insensible_) is:

$$M_{\mathrm{insensible}}=\sum_{j=1}^{m}k_{j}N_{j}$$

Since

$$N_{\mathrm{insensible}}=\sum_{j=1}^{m}N_{j}$$

an effective nitrogen-to-material mass conversion factor can be defined as follows:

$$k_{\mathrm{eff}}=\frac{\sum_{j=1}^{m}k_{j}N_{j}}{\sum_{j=1}^{m}N_{j}}=\frac{M_{\mathrm{insensible}}}{N_{\mathrm{insensible}}},$$

such that

*M*_insensible_ = *k*_eff_*N*_insensible_

This formulation does not require the chemically heterogeneous components of non-urinary nitrogen loss to share a common conversion factor. Rather, *k*_eff_ represents their aggregate material-to-nitrogen mass ratio. Notably, *k*_eff_ > 0 for any physically admissible composition of nitrogen-containing material.

Finally, the role of obligatory non-urinary nitrogen-containing material in the present analysis requires three important distinctions:

- Obligatory non-urinary nitrogen-containing material is not a body substrate that serves as a metabolic fuel [20,21,22,23]. Rather, it represents nitrogen-containing material that is physically eliminated from the body and therefore contributes directly to body mass outflow.
- This material is not itself a product of energy production [20,21,22,23]. Its elimination represents a material flux and should therefore not be interpreted as an additional energetic output associated with substrate oxidation.
- The metabolic energy associated with the physiological handling of this material is already accounted for in energy expenditure [26,29,30,35,36,37]. Its production, processing, transport, and elimination may require adenosine triphosphate (ATP), but these energetic costs are already represented in *EE* through the oxidation of carbohydrate, fat, and protein used, directly or indirectly, to supply the required ATP.

Accordingly, *N*_insensible_ is not included as an independent component of measured energy expenditure [26,29,30,35,36,37]. Introducing an additional energy expenditure term specifically to represent the energetic cost of producing, processing, transporting, or eliminating this material would therefore count energy already represented by the corresponding substrate-oxidation fluxes a second time. In contrast, the mass *k*_eff_*N*_insensible_ physically leaves the body and therefore constitutes a genuine component of daily mass outflow and body weight dynamics. The mass of the eliminated material and the metabolic energy required for its physiological handling are therefore distinct physical quantities that enter different accounting relationships: the former contributes to body mass outflow, whereas the latter contributes to energy expenditure.

### 3.5. Formulation of the Coupled Mass- and Energy-Accounting System at the Coincidence of Energy Balance and Body Weight Stability

As established in Section 3.2, the coincidence of energy balance (*EI* = *EE*) and body weight stability (*dBW*/*dt* = 0) predicted by the EBM necessarily corresponds, by conservation of mass, to whole-body mass equilibrium. Section 3.3 further demonstrated that, at this equilibrium, both fat mass and fat-free mass are stable (*dFM*/*dt* = 0 and *dFFM*/*dt* = 0), thereby providing a direct compartment-level basis for the requirement that the long-term mean nitrogen balance must equal zero. Section 3.4 then formulated this nitrogen-homeostasis requirement quantitatively and distinguished the nitrogen flux *N*_insensible_ from the mass *k*_eff_*N*_insensible_ of the material through which this nitrogen is eliminated. These results provide the basis for formulating the coupled mass- and energy-accounting system.

Accordingly, the required accounting system can be stated in words as follows:

Mass Inflow (g/day) = Mass Outflow (g/day)

Energy Intake (kcal/day) = Energy Expenditure (kcal/day)

Total mass inflow (*M*_in_) includes water intake from beverages and food (*WI*), O_2_ mass uptake associated with metabolic oxidation (*m*_O2_), absorbed carbohydrate intake (*CI_a_*), absorbed protein intake (*PI_a_*), absorbed fat intake (*FI_a_*), and the aggregate inflow of other non-nitrogen-containing material (*M*_other, in_):

(10) $$M_{\mathrm{in}}=\mathrm{WI}+m_{O_{2}}+FI_{a}\;+\;CI_{a}+\;PI_{a}+M_{\mathrm{other},\;\mathrm{in}}.$$

Total mass outflow (*M*_out_) is represented by the mass of generated oxidation products (*m*_ox_), the mass of non-urinary nitrogen-containing material (*k*_eff_*N*_insensible_), water loss (*WL*) not contained within the oxidation products, and the aggregate outflow of other non-nitrogen-containing material (*M*_other, out_):

*M*_out_ = *m*_ox_ +  *k*_eff_*N*_insensible_ + *WL* + *M*_other, out_.

The *M*_other_ terms are introduced solely to ensure complete material accounting for fluxes not represented by the preceding terms. They exclude nitrogen-containing material already represented by the protein and *N*_insensible_-associated fluxes.

Substrate oxidation obeys the following generalized chemical relationship [29,30,35,36,37,38]:

$$\mathrm{FO}x_{\mathrm{net}}\;+\;\;\mathrm{CO}x_{\mathrm{net}}\;+\;\;\mathrm{PO}x_{\mathrm{net}}+m_{O_{2}}\to \;\;m_{\mathrm{ox}}\;+\;\mathrm{Heat}$$

where *COx_net_*, *POx_net_*, and *FOx_net_* denote net carbohydrate, protein, and fat oxidation, respectively. Because the atoms present in the oxidation products originate from the reactants, the principle of mass conservation requires that

$$m_{\mathrm{ox}}=\mathrm{FO}x_{\mathrm{net}}\;+\;\;\mathrm{CO}x_{\mathrm{net}}\;+\;\;\mathrm{PO}x_{\mathrm{net}}+m_{O_{2}},$$

implying that

(11) $$M_{\mathrm{out}}=\mathrm{FO}x_{\mathrm{net}}\;+\;\;\mathrm{CO}x_{\mathrm{net}}\;+\;\;\mathrm{PO}x_{\mathrm{net}}+\;\;m_{O_{2}}+\;\;k_{\mathrm{eff}}N_{\mathrm{insensible}}+\mathrm{WL}+M_{\mathrm{other},\;\mathrm{out}}.$$

Here, net carbohydrate oxidation is defined as total carbohydrate oxidation minus gluconeogenesis plus de novo lipogenesis, since gluconeogenesis generates carbohydrate mass whereas de novo lipogenesis removes carbohydrate mass through conversion into lipid [29,30,38].

Energy intake (*EI*) is determined by the energy-containing macronutrient inflows *CI_a_*, *PI_a_*, and *FI_a_* after scaling each by its respective energy density [29,30,35,36,37,38]:

*EI* = *ρ_F_FI_a_* + *ρ_C_CI_a_* + *ρ_P_PI_a_* (12)

where *ρ_X_* is the energy density of macronutrient *X* (kcal/g). Similarly, energy expenditure (*EE*) is determined by the corresponding net oxidative macronutrient fluxes *COx_net_*, *POx_net_*, and *FOx_net_* [29,30,35,36,37,38]:

*EE* = *ρ_F_FOx_net_*  +   *ρ_C_COx_net_*  +   *ρ_P_* *POx_net_*. (13)

Observe that Equation (13) contains no separate energy expenditure term associated with *N*_insensible_. As established in Section 3.4, such a term is unnecessary because the nitrogen-containing material through which *N*_insensible_ is eliminated is neither a metabolic fuel nor itself a product of energy production [20,21,22,23]. Moreover, any metabolic energy required for the production, processing, transport, and elimination of this material is already implicitly accounted for in *EE* through the oxidation of substrates used, directly or indirectly, to supply the required ATP [26,29,30,35,36,37]. Consequently, introducing an additional energy expenditure term specifically associated with *N*_insensible_ would count energy already represented in EE a second time. In contrast, the mass *k*_eff_*N*_insensible_ physically leaves the body and therefore must be explicitly included in the mass outflow equation. Thus, *N*_insensible_ requires an explicit material term in the mass accounting but no additional energetic term in the energy accounting.

At the prolonged physiological steady state considered here, neither body water nor the body stores associated with the material represented by *M*_other_ can undergo sustained net accumulation or depletion. Their long-term mean inflows and outflows must therefore satisfy

*WI* = *WL*

and

*M*_other, in_ = *M*_other, out_

Substituting Equations (10)–(13) into the accounting system and applying these steady state relationships yields:

(14) $$\begin{array}{c} FI_{a}\;+\;CI_{a}+PI_{a}=\mathrm{FO}x_{\mathrm{net}}+\mathrm{CO}x_{\mathrm{net}}+\mathrm{PO}x_{\mathrm{net}}+k_{\mathrm{eff}}N_{\mathrm{insensible}}\\ \rho_{F}FI_{a}+\rho_{C}CI_{a}+\rho_{P}PI_{a}=\rho_{F}\mathrm{FO}x_{\mathrm{net}}+\rho_{C}\mathrm{CO}x_{\mathrm{net}}+\rho_{P}\;\mathrm{PO}x_{\mathrm{net}} \end{array}$$

The solution to system (14) is derived in the next section.

### 3.6. The Solution to System (14) Determines the Intake-Oxidation Relationships Associated with the Coincidence of Energy Balance and Body Weight Stability

Solving system (14) requires selecting a set of unknown variables while treating the remaining quantities as specified. Importantly, as demonstrated in Appendix A.5, the solution obtained from the system does not depend on which variables are selected as unknowns. For the present derivation, *FI_a_*, *CI_a_*, and *PI_a_* are therefore chosen as the unknown variables, with all other quantities regarded as known.

The system can be simplified further using the relationship previously established for the coincidence of energy balance and body weight stability (see Section 3.3):

*FI_a_* = *FOx_net_*

Substitution of this relationship into (14) eliminates the fat terms from both accounting equations, reducing the system to:

(15) $$\begin{array}{c} CI_{a}+\;PI_{a}=\mathrm{CO}x_{\mathrm{net}}+\mathrm{PO}x_{\mathrm{net}}+k_{\mathrm{eff}}N_{\mathrm{insensible}}\\ \rho_{C}CI_{a}+\;\rho_{P}PI_{a}=\;\rho_{C}\mathrm{CO}x_{\mathrm{net}}+\rho_{P}\;\mathrm{PO}x_{\mathrm{net}.} \end{array}$$

Accordingly, system (14) has the solution:

$$\begin{array}{c} FI_{a}=\mathrm{FO}x_{\mathrm{net}},\\ CI_{a}\;=c,\\ PI_{a}=\;p \end{array}$$

where *c* and *p* are determined by solving system (15).

Atwater-based approximations commonly simplify the analysis by treating *ρ_C_* and *ρ_P_* as equal, whereas values derived from metabolic calorimetry indicate that their energy densities differ [36,37,38] (e.g., *ρ_C_* ≈ 4.2 kcal/g, *ρ_P_* ≈ 4.7 kcal/g [38]). This distinction is analytically important because setting *ρ_C_* = *ρ_P_* causes system (15) to admit solutions only when

*N*_insensible_ = 0

a condition that is incompatible with the fact that obligatory non-urinary nitrogen losses are always greater than zero (see Equation (8)) [20,21].

To demonstrate this result, note that if *ρ_C_* = *ρ_P_*, then the energy densities in system (15) cancel out, yielding:

$$\begin{array}{c} CI_{a}\;+\;PI_{a}=\mathrm{CO}x_{\mathrm{net}}+\mathrm{PO}x_{\mathrm{net}}+k_{\mathrm{eff}}N_{\mathrm{insensible}}\\ CI_{a}\;+\;PI_{a}=\mathrm{CO}x_{\mathrm{net}}+\mathrm{PO}x_{\mathrm{net}.} \end{array}$$

Subtracting the lower equation from the upper equation gives:

*k*_eff_*N*_insensible_ = 0

Therefore, since *k*_eff_ > 0, it follows that the only possible solution is:

*N*_insensible_ = 0,

which completes the demonstration.

Since *ρ_C_* ≠ *ρ_P_*, system (15) can be expressed in matrix form as follows:

$$\left[\begin{array}{cc} 1 & 1\\ \rho_{C} & \rho_{P} \end{array}\right]\left[\begin{array}{c} CI_{a}\\ PI_{a} \end{array}\right]=\left\{\begin{array}{c} \mathrm{CO}x_{\mathrm{net}}+\mathrm{PO}x_{\mathrm{net}}+k_{\mathrm{eff}}N_{\mathrm{insensible}}\\ \rho_{C}\mathrm{CO}x_{\mathrm{net}}+\rho_{P}\mathrm{PO}x_{\mathrm{net}} \end{array}\right\}$$

Solving this matrix equation by inversion gives:

$$\begin{array}{cc} \left[\begin{array}{c} CI_{a}\\ PI_{a} \end{array}\right] & =\frac{1}{\rho_{P}-\rho_{C}}\left[\begin{array}{cc} \rho_{P} & -1\\ -\rho_{C} & 1 \end{array}\right]\left\{\begin{array}{c} \mathrm{CO}x_{\mathrm{net}}+\mathrm{PO}x_{\mathrm{net}}+k_{\mathrm{eff}}N_{\mathrm{insensible}}\\ \rho_{C}\mathrm{CO}x_{\mathrm{net}}+\rho_{P}\mathrm{PO}x_{\mathrm{net}} \end{array}\right\}\\ & =\left[\begin{array}{c} \mathrm{CO}x_{\mathrm{net}}+\left(\frac{\rho_{P}}{\rho_{P}-\rho_{C}}\right)k_{\mathrm{eff}}N_{\mathrm{insensible}}\\ \mathrm{PO}x_{\mathrm{net}}-\left(\frac{\rho_{C}}{\rho_{P}-\rho_{C}}\right)k_{\mathrm{eff}}N_{\mathrm{insensible}} \end{array}\right] \end{array}$$

Consequently, the complete solution of system (14) is:

$$\begin{array}{c} FI_{a}=\mathrm{FO}x_{\mathrm{net}},\\ CI_{a}\;=c=\mathrm{CO}x_{\mathrm{net}}+\left(\frac{\rho_{P}}{\rho_{P}-\rho_{C}}\right)k_{\mathrm{eff}}N_{\mathrm{insensible}},\\ PI_{a}=\;p=\mathrm{PO}x_{\mathrm{net}}-\left(\frac{\rho_{C}}{\rho_{P}-\rho_{C}}\right)k_{\mathrm{eff}}N_{\mathrm{insensible}} \end{array}$$

The physiological interpretation of this solution is that, according to the EBM analytical framework, any macronutrient flux configuration that results in the simultaneous occurrence of energy balance and body weight stability must obey the derived intake-oxidation relationships because no alternative solution is compatible with the internal analytical structure of the EBM.

To verify that this set of equations indeed generates the coincidence of energy balance and body weight stability, it is sufficient to demonstrate that substituting the solution to system (14) into Equation (12) transforms the expression into Equation (13):

$$\begin{array}{cc} \mathrm{EI} & =\rho_{F}FI_{a}+\;\rho_{C}CI_{a}+\;\rho_{P}PI_{a}\\ & =\rho_{F}\mathrm{FO}x_{\mathrm{net}}\;+\;\rho_{C}\left[\mathrm{CO}x_{\mathrm{net}}+\left(\frac{\rho_{P}}{\rho_{P}-\rho_{C}}\right)k_{\mathrm{eff}}N_{\mathrm{insensible}}\right]\;+\;\rho_{P}\left[\mathrm{PO}x_{\mathrm{net}}-\left(\frac{\rho_{C}}{\rho_{P}-\rho_{C}}\right)k_{\mathrm{eff}}N_{\mathrm{insensible}}\right]\\ & =\rho_{F}\mathrm{FO}x_{\mathrm{net}}\;+\;\rho_{C}\mathrm{CO}x_{\mathrm{net}}\;+\;\rho_{P}\mathrm{POx}+\;\;\left(\frac{\rho_{C}\rho_{P}}{\rho_{P}-\rho_{C}}\right)k_{\mathrm{eff}}N_{\mathrm{insensible}}-\left(\frac{\rho_{P}\rho_{C}}{\rho_{P}-\rho_{C}}\right)k_{\mathrm{eff}}N_{\mathrm{insensible}}\\ & =\rho_{F}\mathrm{FO}x_{\mathrm{net}}+\;\rho_{C}\mathrm{CO}x_{\mathrm{net}}+\;\rho_{P}\mathrm{PO}x_{\mathrm{net}}\\ & =\mathrm{EE} \end{array}$$

Similarly, substitution of the solution to system (14) into Equation (10), together with the prolonged-equilibrium conditions *WI* = *WL* and *M*_other, in_ = *M*_other, out_ used in formulating (14), transforms the expression into Equation (11):

$$\begin{array}{cc} M_{\mathrm{in}} & =\mathrm{WI}+m_{O_{2}}+\;FI_{a}+CI_{a}+PI_{a}+M_{\mathrm{other},\;\mathrm{in}}\\ & =\mathrm{WL}+m_{O_{2}}+\;\mathrm{FO}x_{\mathrm{net}}+\;\left[\mathrm{CO}x_{\mathrm{net}}+\left(\frac{\rho_{P}}{\rho_{P}-\rho_{C}}\right)k_{\mathrm{eff}}N_{\mathrm{insensible}}\right]\;+\;\left[\mathrm{PO}x_{\mathrm{net}}-\left(\frac{\rho_{C}}{\rho_{P}-\rho_{C}}\right)k_{\mathrm{eff}}N_{\mathrm{insensible}}\right]+M_{\mathrm{other},\;\mathrm{out}}\\ & =m_{O_{2}}+\mathrm{FO}x_{\mathrm{net}}+\mathrm{CO}x_{\mathrm{net}}+\;\mathrm{PO}x_{\mathrm{net}}+\left(\frac{\rho_{P}-\rho_{C}}{\rho_{P}-\rho_{C}}\right)k_{\mathrm{eff}}N_{\mathrm{insensible}}+\mathrm{WL}+M_{\mathrm{other},\;\mathrm{out}}\\ & =\mathrm{FO}x_{\mathrm{net}}+\mathrm{CO}x_{\mathrm{net}}+\mathrm{PO}x_{\mathrm{net}}+\;m_{O_{2}}+k_{\mathrm{eff}}N_{\mathrm{insensible}}+\mathrm{WL}+M_{\mathrm{other},\;\mathrm{out}}\\ & =M_{\mathrm{out}} \end{array}$$

Thus, the derived intake-oxidation relationships simultaneously yield equality of energy intake and energy expenditure and equality of mass inflow and mass outflow. Hence, the solution to system (14) indeed generates the simultaneous conditions of energy balance and mass equilibrium.

### 3.7. Internal Consistency Analysis: Incompatibility of the EBM-Predicted Equilibrium with Nitrogen Homeostasis

The solution to system (14) initially appears internally consistent because it generates the simultaneous occurrence of energy balance and body weight stability, as required by Equation (3). Upon closer examination, however, the solution reveals a striking inconsistency. Because *N*_insensible_ > 0, *k*_eff_ > 0, and *ρ_P_* > *ρ_C_* > 0, this solution necessarily yields

$$\begin{array}{l} CI_{a}\;-\mathrm{CO}x_{\mathrm{net}}=\left(\frac{\rho_{P}}{\rho_{P}-\rho_{C}}\right)k_{\mathrm{eff}}N_{\mathrm{insensible}}>0\\ PI_{a}-\mathrm{PO}x_{\mathrm{net}}=-\left(\frac{\rho_{C}}{\rho_{P}-\rho_{C}}\right)k_{\mathrm{eff}}N_{\mathrm{insensible}}<0 \end{array}$$

Thus, the same solution that predicts energy balance and stable body weight requires a persistent positive carbohydrate balance together with a persistent negative protein balance. If extrapolated indefinitely, these constant flux imbalances imply continuous accumulation of carbohydrate-containing body material and progressive depletion of body protein, ultimately extending the mathematical solution into the physically impossible domain of negative protein mass. The solution therefore preserves total body mass mathematically only through opposing and persistent changes in body constituents and cannot represent a prolonged physiological steady-state.

This inconsistency is also manifested directly through nitrogen balance. Substituting the expression for *PI_a_* obtained from the solution to system (14) into Equation (5) gives:

$$\begin{array}{cc} PI_{a} & =\mathrm{PO}x_{\mathrm{net}}-\left(\frac{\rho_{C}}{\rho_{P}-\rho_{C}}\right)k_{\mathrm{eff}}N_{\mathrm{insensible}}\\ & ⇓\\ \mathrm{NB} & =\frac{PI_{a}-\mathrm{PO}x_{\mathrm{net}}}{k_{P}}\;\;-N_{\mathrm{insensible}}\\ & =\frac{\mathrm{PO}x_{\mathrm{net}}-\left(\frac{\rho_{C}}{\rho_{P}-\rho_{C}}\right)k_{\mathrm{eff}}N_{\mathrm{insensible}}-\mathrm{PO}x_{\mathrm{net}}}{k_{P}}\;\;-\;\;\;N_{\mathrm{insensible}}\\ & =-\left[1+\frac{k_{\mathrm{eff}}\rho_{C}}{k_{P}\left(\rho_{P}-\rho_{C}\right)}\right]N_{\mathrm{insensible}} \end{array}$$

and therefore

*NB* < 0 

because *k_P_* > 0, *k*_eff_ > 0, *ρ_P_* > *ρ_C_* > 0, and *N*_insensible_ > 0.

One might argue, however, that these inconsistencies arise because the solution to (14) was obtained without explicitly enforcing the condition *NB* = 0. As demonstrated in Section 3.3 and Section 3.4, *NB* = 0 is not an additional equilibrium condition imposed on the EBM, but an implicit physiological requirement of the prolonged body weight equilibrium predicted by the framework. The system can therefore be solved again subject to this requirement.

From Equation (5), if nitrogen balance is zero (*NB* = 0), then:

$$N_{\mathrm{insensible}}=\frac{PI_{a}-\mathrm{PO}x_{\mathrm{net}}}{k_{P}}.$$

Thus, enforcing the condition *NB* = 0 in the solution to system (14) means that *N*_insensible_ is no longer an independent quantity. Instead, it is constrained to equal the protein balance, *PI_a_ − POx_net_*, divided by *k_P_*. Consequently, *N*_insensible_ must vary in direct proportion to the protein balance term already present in the mass- and energy-accounting equations.

Substituting *N*_insensible_ = (*PI_a_ − POx_net_*)/*k_P_* directly into the mass balance equation of system (15) yields the following matrix equation:

$$\left[\begin{array}{cc} 1 & \left(1-k_{\mathrm{eff}}/k_{P}\right)\\ \rho_{C} & \rho_{P} \end{array}\right]\left[\begin{array}{c} CI_{a}\\ PI_{a} \end{array}\right]=\left\{\begin{array}{c} \mathrm{CO}x_{\mathrm{net}}+\left(1-k_{\mathrm{eff}}/k_{P}\right)\mathrm{PO}x_{\mathrm{net}}\\ \rho_{C}\mathrm{CO}x_{\mathrm{net}}+\rho_{P}\mathrm{PO}x_{\mathrm{net}} \end{array}\right\}$$

Solving by matrix inversion gives:

$$\begin{array}{cc} \left[\begin{array}{c} CI_{a}\\ PI_{a} \end{array}\right] & =\frac{1}{\rho_{P}-\rho_{C}(1-k_{\mathrm{eff}}/k_{P})}\left[\begin{array}{cc} \rho_{P} & -\left(1-k_{\mathrm{eff}}/k_{P}\right)\\ -\rho_{C} & 1 \end{array}\right]\left\{\begin{array}{c} \mathrm{CO}x_{\mathrm{net}}+\left(1-k_{\mathrm{eff}}/k_{P}\right)\mathrm{PO}x_{\mathrm{net}}\\ \rho_{C}\mathrm{CO}x_{\mathrm{net}}+\rho_{P}\mathrm{PO}x_{\mathrm{net}} \end{array}\right\}\\ & =\left[\begin{array}{c} \mathrm{CO}x_{\mathrm{net}}\\ \mathrm{PO}x_{\mathrm{net}} \end{array}\right] \end{array}$$

Thus, after enforcing *NB* = 0, the solution to system (14) becomes:

$$\begin{array}{c} FI_{a}\;=\mathrm{FO}x_{\mathrm{net}},\\ CI_{a}\;=\mathrm{CO}x_{\mathrm{net}},\\ PI_{a}=\;\mathrm{PO}x_{\mathrm{net}} \end{array}$$

Notice that this constrained solution is independent of the numerical values of the conversion factors *k*_eff_ and *k_P_*, or of their ratio, and it also eliminates the persistent carbohydrate accumulation and protein depletion produced by the unconstrained solution. However, it introduces a second and unavoidable inconsistency because substitution of *PI_a_* = *POx_net_* into Equation (6) yields:

$$N_{\mathrm{insensible}}=\frac{PI_{a}-\mathrm{PO}x_{\mathrm{net}}}{k_{P}}=0,$$

which is physiologically impossible since obligatory non-urinary nitrogen losses cannot vanish [20,21].

More specifically, the enforcement of the implicitly required condition *NB* = 0 generates the simultaneous occurrence of

body weight stability (*dBW*/*dt* = 0),

energy balance (*EI* = *EE*),

nitrogen balance (*NB* = 0),

yet the resulting solution requires net protein oxidation to equal absorbed protein intake, *PI_a_* = *POx_net_*, and therefore, by Equation (6), *N*_insensible_ = 0. Thus, under the constrained solution, nitrogen balance is achieved only when the equality *PI_a_* = *POx_net_* forces obligatory non-urinary nitrogen losses to vanish, a physiologically impossible condition. Furthermore, because *PI_a_* = *POx_net_* makes the numerator of Equation (6) equal to zero, the resulting requirement *N*_insensible_ = 0 is independent of the numerical value assigned to the nitrogen-to-protein conversion factor *k_P_*.

Consequently, the present analysis demonstrates that the EBM accounting framework does not yield a physiologically consistent equilibrium solution under the simultaneous conditions of energy balance and prolonged body weight stability. As established in Section 3.2, Section 3.3 and Section 3.4, the EBM-derived condition *dBW*/*dt* = 0 necessarily corresponds, by conservation of mass, to whole-body mass equilibrium, while the resulting prolonged stability of fat mass and fat-free mass implicitly requires a mean nitrogen balance equal to zero. When this requirement is left implicit, the solution to system (14) maintains stable body weight through persistent carbohydrate accumulation and protein depletion and consequently produces a persistent negative nitrogen balance. When *NB* = 0 is explicitly enforced, these persistent macronutrient imbalances disappear, but the resulting solution instead requires the physiologically impossible condition *N*_insensible_ = 0. Thus, whether the nitrogen homeostasis requirement is left implicit or explicitly enforced, the coupled accounting system fails to produce a physiologically admissible prolonged equilibrium while maintaining the simultaneous EBM conditions of energy balance and body weight stability.

## 4. Discussion

The present theoretical analysis shows that the EBM cannot produce a physiologically consistent prolonged equilibrium that simultaneously satisfies energy balance (*EI* = *EE*), body weight stability (*dBW*/*dt* = 0), and nitrogen homeostasis (*NB* = 0) when obligatory non-urinary nitrogen losses are explicitly recognized.. Importantly, nitrogen balance is not introduced here as an externally imposed equilibrium assumption. As demonstrated in Section 3.2, Section 3.3 and Section 3.4, within the EBM, *EI* = *EE* implies *dBW*/*dt* = 0, and this equilibrium further implies stability of both fat mass and fat-free mass (*dFM*/*dt* = 0 and *dFFM*/*dt* = 0). Because these compartments together constitute total body mass, their prolonged stability requires the absence of sustained accumulation or depletion of nitrogen-containing body material and therefore a mean nitrogen balance equal to zero. Nitrogen homeostasis thus emerges as an implicit physiological requirement of the same prolonged equilibrium predicted by the EBM, providing the basis for testing whether that equilibrium is internally self-consistent.

Explicit examination of this consistency requirement reveals the internal inconsistency in two complementary ways. If the nitrogen homeostasis requirement is left implicit, the unconstrained coupled mass- and energy-accounting system preserves energy balance and stable body weight only through a persistent positive carbohydrate balance and a persistent negative protein balance, thereby producing a sustained negative nitrogen balance. Conversely, when the implicitly required condition *NB* = 0 is explicitly enforced, these persistent macronutrient imbalances disappear, but the resulting solution requires *N*_insensible_ = 0, despite obligatory non-urinary nitrogen losses being physiologically unavoidable. Thus, neither the unconstrained nor the nitrogen constrained solution provides a physiologically admissible prolonged equilibrium.

These findings do not arise from omission of an energetic cost associated with non-urinary nitrogen elimination. As established in Section 3.4, the nitrogen-containing material through which *N*_insensible_ is eliminated is neither a metabolic fuel nor itself a product of energy production [20,21,22,23], and any metabolic energy required for its production, processing, transport, and elimination is already accounted for within *EE* through the oxidation of substrates supplying the required metabolic work [26,29,30,35,36,37,38]. The inconsistency therefore arises because, under the simultaneous equilibrium conditions examined here, the canonical EBM cannot reconcile energy balance with the material and nitrogen-homeostasis requirements of prolonged body weight stability. Accordingly, the discussion below considers why this incompatibility may have remained unrecognized and examines its implications for body weight regulation and for the distinction between energetic and material descriptions of metabolism.

### 4.1. Why the Inconsistency May Have Remained Unrecognized

Several factors may explain why the inconsistency identified in the present analysis has received limited attention within the energy balance literature. First, prolonged body weight stability is inherently difficult to study experimentally because establishing a physiological steady state requires sufficiently long observation periods to distinguish short-term fluctuations in body weight from sustained changes in body mass. Consequently, most experimental and theoretical work has focused on transient states of weight gain or weight loss rather than on the physical and physiological requirements of prolonged equilibrium itself [10,11,12,13,14,27,29].

A further reason the inconsistency may have remained unnoticed is that much of the contemporary discussion of body weight regulation has centered on the physiological mechanisms controlling energy intake, energy expenditure, appetite, substrate utilization, and neuroendocrine regulation [39,40,41]. Although competing interpretations differ substantially regarding the mechanisms responsible for changes in food intake, expenditure, and body composition, these debates generally operate within an underlying framework in which the energetic accounting relationships themselves are taken as the starting point [39,40]. Consequently, comparatively less attention has been directed toward asking whether the prolonged equilibrium generated by those equations remains internally compatible with the requirements of whole-body mass conservation and constituent-specific material homeostasis, which are independent of the EBM framework [24,25,42]. The present analysis addresses this latter question rather than the physiological mechanisms determining how energy intake and expenditure are regulated.

Finally, in practical nutrition research, the governing equations of the EBM are commonly used operationally to estimate energetic quantities and describe changes in body energy stores and body composition [27,29,41]. However, their empirical usefulness does not guarantee that the prolonged equilibrium predicted by these equations satisfies all material requirements of body mass stability. Thus, the issue identified here concerns the internal compatibility of the prolonged equilibrium conditions rather than the validity of conservation of energy or the usefulness of energetic accounting for describing metabolic processes.

### 4.2. Implications for Body Weight Regulation

As mathematically demonstrated in the present analysis, the analytical structure of the EBM implies that energy balance (*EI* = *EE*) leads to body weight stability (*dBW*/*dt* = 0). However, following this EBM-predicted equilibrium to its physiological consequences produces contradictions. The unconstrained solution to system (14) requires a persistent negative protein balance and negative nitrogen balance, implying continuous depletion of nitrogen-containing body material, while simultaneously requiring a persistent positive carbohydrate balance. These fluxes are incompatible with the same EBM-predicted equilibrium, which requires both fat mass and fat-free mass to remain stable. Moreover, if the solution were extrapolated indefinitely, body protein would progressively approach depletion and could no longer provide the continuing mass loss required to offset the persistent carbohydrate accumulation. Carbohydrate accumulation would then continue unopposed, producing body weight gain rather than the body weight stability predicted by the EBM. Conversely, explicit enforcement of nitrogen homeostasis eliminates these persistent macronutrient imbalances only by requiring *N*_insensible_ = 0, which is physiologically impossible because obligatory non-urinary nitrogen losses cannot vanish. These contradictions are therefore not produced by externally forcing energy balance and body weight stability to coexist; their coexistence is itself a mathematically derived consequence of the EBM. Taken together, these results conform to the logical structure of a proof by contradiction or *reductio ad absurdum*. If deductive reasoning from a proposition assumed to be true necessarily leads to a contradiction, that proposition must be false and its negation must be true [43,44]. Applied here, if the proposition that energy balance must correspond to prolonged body weight stability is shown to produce a physiological contradiction, then its negation must be accepted. That is, the present analysis implies that prolonged body weight stability does not coincide with energy balance (*EI* = *EE*) but instead coexists with a state of energy imbalance (*EI* ≠ *EE*).

A practical consequence of this conclusion may already be reflected in empirical studies reporting apparently persistent negative energy balance in individuals maintaining relatively stable body weight. Such observations are commonly attributed to systematic underreporting of energy intake [45,46,47,48]. Although this explanation is plausible within the EBM framework, the present analysis raises the possibility that the apparent mismatch may not arise exclusively from measurement error but may also reflect the fact that energy intake and energy expenditure need not be tightly coupled. Energy expenditure is closely associated with the organism’s metabolic state and patterns of substrate utilization [49,50,51,52]. In contrast, energy intake may be influenced not only by physiological energy requirements but also, and in many circumstances strongly, by food availability, palatability, and the hedonic properties of the food environment [53,54,55,56]. These distinct determinants suggest that energy intake and energy expenditure may vary semi-independently because the factors governing intake during weight stability are not necessarily the same as those determining energy expenditure under the same physiological condition.

Another implication of the mathematical conclusion that body weight stability must coexist with energy imbalance is that energy imbalance cannot, by itself, serve as a sufficient descriptor of body weight dynamics. The relevance of this theoretical conclusion is strengthened by previously reported empirical tests of Equation (3). In a recent analysis of tightly controlled randomized metabolic-ward data from Hall et al. [24], Equation (3) was evaluated using independently measured energy intake, energy expenditure, body composition, and body weight changes. During the initial 14-day phase of the trial (Figure 2 in [24]), energy intake, energy expenditure, and body mass energy density were comparable between the low-carbohydrate (LC) and low-fat (LF) conditions, with modest positive energy balances in both treatments (LC: ~+38.5 kcal/day; LF: ~+231 kcal/day). Equation (3) therefore predicted small increases in body weight under both diets (LC prediction: +0.078 kg; LF prediction: +0.498 kg). The observed responses, however, were markedly different and contrary to the EBM prediction: the LC group lost approximately 2.81 kg of body weight, including about 0.69 kg of fat mass, whereas the LF group lost about 0.647 kg of body weight and about 0.42 kg of fat mass. During the final 14-day phase of the trial (Figure 3 in [24]), the LC condition exhibited a substantial positive measured energy balance (~+1000 kcal/day), for which Equation (3) predicted a body weight gain of approximately 1.68 kg, yet the observed mean body weight change was approximately –0.56 kg. Thus, the discrepancies involved not only the magnitude but also the direction of body weight change predicted by the EBM. Beyond these group-average comparisons, the individual-level analysis shown in Figure 5 of [24] revealed that, after correction for unabsorbed energy intake, 6 of the 12 14-day intervals characterized by positive energy balance were nevertheless associated with body weight loss, with reductions ranging from approximately 0.9 to 2.7 kg. Hence, half of the observed intervals with positive energy balance exhibited a direction of body weight change opposite to that predicted by the EBM. A sensitivity analysis of the LC condition further showed that reconciling the the observed body weight response with Equation (3) would required substantial changes in the measured energy imbalance rather than small corrections within the range of ordinary measurement uncertainty, further supporting the conclusion that energy imbalance alone is insufficient to describe the observed body weight dynamics [24].

Taken together, these findings do not challenge conservation of energy or the physiological importance of energy metabolism. Rather, they indicate that energetic accounting is not, by itself, sufficient to provide a physiologically self-consistent description of the dynamics or equilibrium of a material variable such as body weight. The combined theoretical and empirical findings therefore provide reason to re-examine whether energy balance alone can serve as a sufficient governing description of body weight dynamics. This distinction is examined further in Section 4.3.

### 4.3. Material Basis of Body Weight Dynamics and the Non-Uniqueness of Energetic Descriptions

In energy metabolism, both energy intake and energy expenditure are quantities derived from underlying material fluxes: each is expressed as a weighted sum of macronutrient mass fluxes, with the corresponding energy densities serving as weighting factors (see Equations (12) and (13)) [27,29,30,35,36,37,38]. The same value of energy intake or energy expenditure can therefore correspond to different amounts of absorbed or oxidized material, respectively. Consequently, energetic quantities do not uniquely determine the material fluxes from which they are derived, whereas those material fluxes directly determine changes in total body mass through conservation of mass. Consistent with this distinction, a previous analysis of the Hall et al. data [24] showed that differences in total food mass intake corresponded more closely to the observed body weight and body composition responses than differences in energy intake: during the initial phase, similar energy intakes but markedly different mass intakes were associated with different weight and fat-mass changes, whereas during the final phase, substantially different energy intakes but similar mass intakes were accompanied by similar weight changes.

This non-unique relationship between metabolic energy and mass can be illustrated quantitatively by examining energy expenditure and the mass elimination associated with substrate oxidation. According to the indirect calorimetry equations commonly employed in energy balance research [27,29], energy expenditure (*EE*) and the corresponding rate of metabolically associated material elimination (*M_EE_*) are given by:

$$\begin{array}{c} M_{\mathrm{EE}}=-1.47\times VO_{2}+2.82\times \mathrm{VC}O_{2}+0.73\times N_{\mathrm{urine}}\\ \mathrm{EE}=3.88\times VO_{2}+1.08\times \mathrm{VC}O_{2}-1.52\times N_{\mathrm{urine}} \end{array}$$

where VO_2_ and VCO_2_ denote the volumes of oxygen consumed and carbon dioxide produced, respectively (a derivation of the general mathematical form of these equations is provided in Appendix A.6). To illustrate this non-uniqueness under physiologically realistic conditions, the numerical values in the following examples were selected to represent plausible 24 h metabolic states. In particular, the corresponding respiratory quotients (VCO_2_/VO_2_ = 0.849 and 0.725) fall within the physiological range associated with different mixtures of oxidized substrates [29]. The examples are therefore not intended to represent specific individuals, but rather to demonstrate that the same energy expenditure can arise from different underlying material fluxes under physiologically plausible conditions.

Consider the following two hypothetical metabolic states:

Subject-A: VO_2_ = 525 L/day; VCO_2_ = 445.6 L/day; VCO_2_/VO_2_ = 0.849; *N*_urine_ = 12 g/day.

$$\begin{array}{cc} M_{\mathrm{EE}} & =-1.47\times 525+2.82\times 445.6+0.73\times 12=494\;g/\mathrm{day}\\ \mathrm{EE} & =3.88\times 525+1.08\times 445.6-1.52\times 12=2500\;\mathrm{kcal}/\mathrm{day} \end{array}$$

Subject-B: VO_2_ = 540L/day; VCO_2_ = 391.71 L/day; VCO_2_/VO_2_ = 0.725; *N*_urine_ = 12 g/day

$$\begin{array}{cc} M_{\mathrm{EE}} & =-1.47\times 540+2.82\times 391.71+0.73\times 12=320\;g/\mathrm{day}\\ \mathrm{EE} & =3.88\times 540+1.08\times 391.71-1.52\times 12=2500\;\mathrm{kcal}/\mathrm{day} \end{array}$$

These two hypothetical metabolic states yield the same calculated daily energy expenditure while corresponding to different substrate-oxidation fluxes and different quantities of metabolically associated material elimination. In this example, the calculated rate of material elimination in Subject-A is 54% greater than that in Subject-B. This comparison clearly illustrates that, under physiologically plausible conditions, the same energy expenditure can correspond to different underlying substrate masses. Thus, knowledge of energy expenditure alone does not uniquely specify the amount of material processed and eliminated through metabolism.

Collectively, the present theoretical and quantitative analyses indicate that body weight dynamics are directly constrained by conservation of mass, whereas energetic quantities obtained through calorimetry provide derived descriptions of the underlying metabolic material fluxes. Viewed in this way, the inconsistency identified within the EBM framework may originate from treating energy imbalance as sufficient to determine the material imbalance governing body mass dynamics [24,25,42].

## 5. Conclusions

The internal structure of the EBM predicts that energy balance (*EI* = *EE*) must coexist with body weight stability. However, the logical consequences of this equilibrium state contradict the nitrogen homeostasis required under prolonged body weight stability. Accordingly, by the principles of formal analytical reasoning, the negation of the EBM-derived conclusion must be accepted: body weight stability does not coincide with energy balance (*EI* = *EE*) but instead coexists with a state of energy imbalance (*EI* ≠ *EE)*. This theoretical conclusion is consistent with empirical observations of relatively stable body weight despite apparent persistent energy imbalance and with recent randomized metabolic-ward data showing body weight changes whose direction and magnitude were not correctly predicted by measured energy imbalance [24]. More generally, these findings suggest that energy imbalance alone cannot serve as a sufficient descriptor of body weight dynamics. A possible physical basis for these results is the non-unique correspondence between metabolic energy and the underlying substrate masses: the same energy intake or expenditure can arise from different material fluxes, whereas changes in body weight are directly determined by the balance of those material fluxes through conservation of mass.

## Figures and Tables

**Figure 1:**
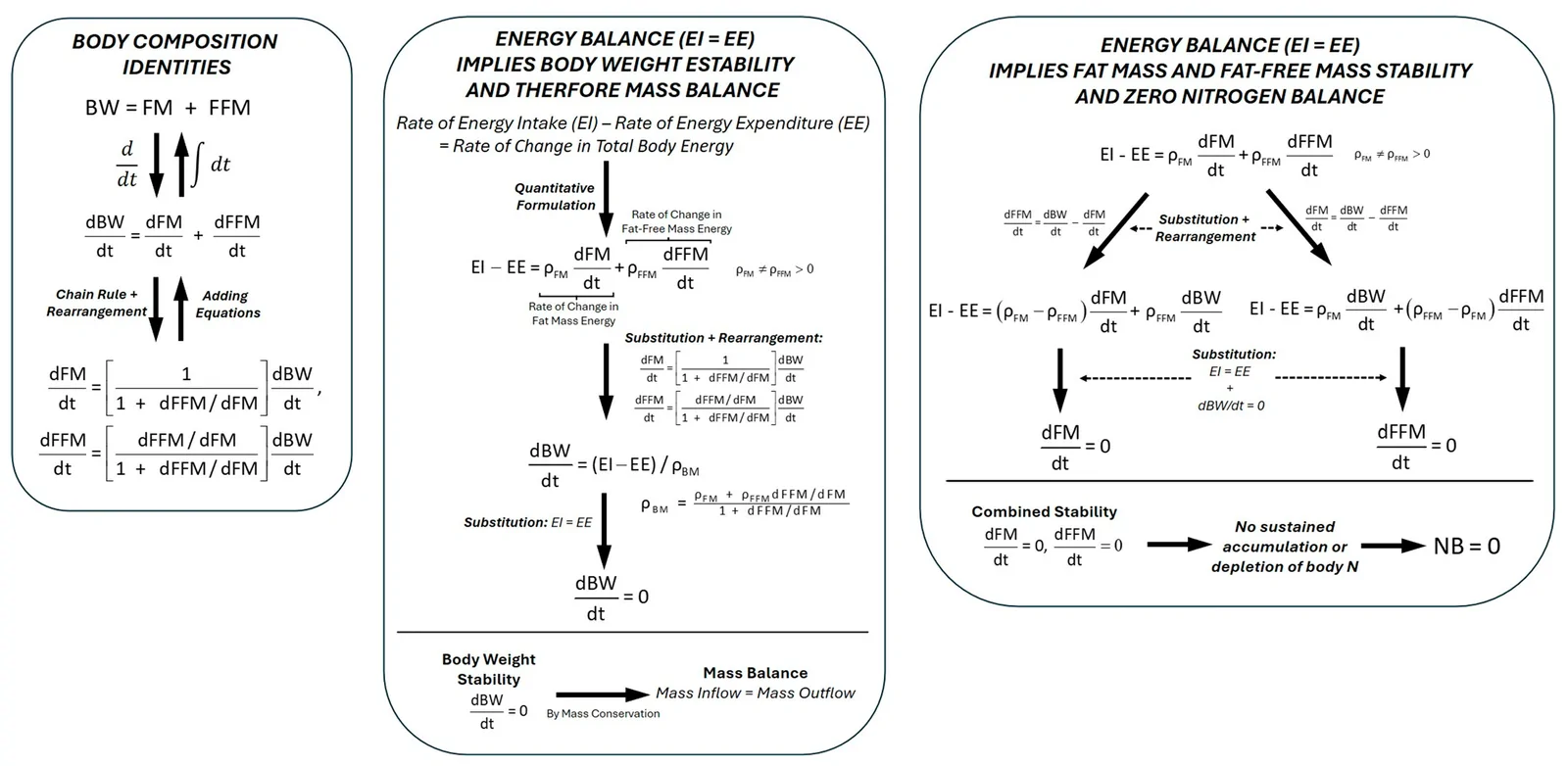
Analytical derivation of the equilibrium conditions implicit in the EBM framework. The left panel presents the body composition identity *BW* = *FM* + *FFM* and the corresponding relationships among the rates of change in body weight (*BW*), fat mass (*FM*), and fat-free mass (*FFM*). As indicated in the figure, these identities follow directly from the definition of body composition and are independent of the EBM framework. The middle panel incorporates these model-independent identities into the canonical EBM equation (Equation (1)), which represents total body energy as the sum of the energy stored in fat mass and fat-free mass and relates the difference between energy intake (*EI*) and energy expenditure (*EE*) to the rate of change of these two energy stores. This derivation yields *dBW*/*dt* = (*EI* − *EE*)/*ρ_BM_*, where *ρ_BM_* > 0, demonstrating that, within the EBM framework, energy balance (*EI* = *EE*) implies body weight stability (*dBW*/*dt* = 0). Because body weight represents body mass, conservation of mass further requires that this body weight steady-state correspond to equality between total mass inflow and total mass outflow. The right panel substitutes alternative rearrangements of the body composition identity into Equation (1) to derive the corresponding compartment-level consequences of this equilibrium. Under the simultaneous conditions *EI* = *EE* and *dBW*/*dt* = 0, and because *ρ_FM_ ≠ ρ_FFM_* > 0, the resulting equations require *dFM*/*dt* = 0 and *dFFM*/*dt* = 0. Thus, both fat mass and fat-free mass are stable at the EBM-predicted equilibrium. Because these compartments together constitute total body mass, their combined stability precludes sustained accumulation or depletion of nitrogen-containing body material and therefore requires a zero long-term mean nitrogen balance (*NB* = 0). Thus, the figure shows that the EBM-predicted coincidence of energy balance and body weight stability necessarily entails simultaneous body mass, body composition, and nitrogen equilibrium.

## Data Availability

The original contributions presented in this study are included in the article. Further inquiries can be directed to the corresponding author.

## Abbreviations

The following abbreviations are used in this manuscript:

ATP	Adenosine Triphosphate
*BM*	Body Mass
*BW*	Body Weight
*CI_a_*	Absorbed Carbohydrate Intake
CO_2_	Carbon Dioxide
*COx_net_*	Net Carbohydrate Oxidation
DNL	De Novo Lipogenesis
EBM	Energy Balance Model
*EE*	Energy Expenditure
*EI*	Energy Intake
FFM	Fat-Free Mass
*FI_a_*	Absorbed Fat Intake
FM	Fat Mass
*FOx_net_*	Net Fat Oxidation
GNG	Gluconeogenesis
*k*_eff_	Effective Nitrogen-to-Material Mass conversion factor
*k_P_*	Nitrogen-to-Protein Conversion Factor
*M*_in_	Mass Inflow
*M*_insensible_	Total Mass of Non-Urinary Nitrogen-Containing Material Eliminated
*m*_O2_	Inhaled Oxygen Mass Associated with Metabolic Oxidation
*M*_other, in_	Aggregate Mass Inflow of Other Non-Nitrogen-Containing Material
*M*_other, out_	Aggregate Mass Outflow of Other Non-Nitrogen-Containing Material
*M_out_*	Mass Outflow
*m*_ox_	Mass of Oxidation Products
*NB*	Nitrogen Balance
*N_in_*	Dietary Nitrogen Intake
*N*_insensible_	Obligatory Non-Urinary Nitrogen Losses
*N*_urine_	Urinary Nitrogen Excretion
O_2_	Oxygen
*PI_a_*	Absorbed Protein Intake
*POx_net_*	Net Protein Oxidation
*ρ_BM_*	Body Mass Energy Density
*ρ_C_*	Energy Density of Carbohydrate
*ρ_F_*	Energy Density of Fat
*ρ_FFM_*	Fat-Free Mass Energy Density
*ρ_FM_*	Fat Mass Energy Density
*ρ_P_*	Energy Density of Protein
VCO_2_	Carbon Dioxide Production
VO_2_	Oxygen Consumption
*WI*	Water Intake from Beverages and Food
*WL*	Water Loss Not Contained Within The Oxidation Products

## Appendix A

### Appendix A.1. Derivation of Equation (1)

Total body energy (TBE, in kcal) consists of the energy stored in fat mass (FME) and fat-free mass (FFME) [26]:

*TBE* = *FME* + *FFME*

For each body compartment, stored energy is obtained by multiplying its mass by its corresponding energy density. Accordingly,

*FME* = *ρ_FM_FM*,  *FFME* = *ρ_FFM_FFM*,

where *FM* and *FFM* denote fat mass and fat-free mass (kg), respectively, and *ρ_FM_* and *ρ_FFM_* denote their respective energy densities (kcal/kg), treated as characteristic constants of the corresponding body compartments [13,27,28]. Substitution into the expression for total body energy gives:

*TBE* = *ρ_FM_FM* + *ρ_FFM_FFM*

Taking the derivative of TBE with respect to time (days) yields:

$$\frac{\mathrm{dTBE}}{\mathrm{dt}}=\rho_{\mathrm{FM}}\frac{\mathrm{dFM}}{\mathrm{dt}}+\rho_{\mathrm{FFM}}\frac{\mathrm{dFFM}}{\mathrm{dt}}$$

Energy conservation requires the rate of change in total body energy to equal the difference between energy intake (*EI*, kcal/day) and energy expenditure (*EE*, kcal/day) [26]. Therefore,

$$\frac{\mathrm{dTBE}}{\mathrm{dt}}=\mathrm{EI}-\mathrm{EE}.$$

Equating the two expressions for the rate of change in total body energy gives Equation (1):

$$\mathrm{EI}-\mathrm{EE}=\rho_{\mathrm{FM}}\frac{\mathrm{dFM}}{\mathrm{dt}}+\rho_{\mathrm{FFM}}\frac{\mathrm{dFFM}}{\mathrm{dt}}.$$

### Appendix A.2. Derivation of Equation (2)

Within the energy balance framework, increases in fat mass arise from absorbed dietary fat (*FI_a_*, g/day) together with lipid synthesized through de novo lipogenesis (*DNL*, g/day):

Fat Accumulation = *FI_a_* + *DNL*

Fat mass is simultaneously depleted through fat oxidation (*FOx*, g/day):

Fat Loss = *FOx*

The daily rate of change in fat mass therefore corresponds to the difference between these opposing fluxes [29]:

$$\begin{array}{cc} \frac{\mathrm{dFM}}{\mathrm{dt}} & =\left(\mathrm{Fat}\;\mathrm{Accumulation}\right)-\left(\mathrm{Fat}\;\mathrm{Loss}\right)\\ & =FI_{a}+\mathrm{DNL}-\mathrm{FOx}\\ & =FI_{a}-\left(\mathrm{FOx}-\mathrm{DNL}\right) \end{array}$$

Defining net fat oxidation (*FOx_net_*) as

*FOx_net_* = *FOx* − *DNL*,

allows the preceding relationship to be expressed as follows:

$$\frac{\mathrm{dFM}}{\mathrm{dt}}=FI_{a}-\mathrm{FO}x_{\mathrm{net}}$$

which corresponds to Equation (2).

### Appendix A.3. Derivation of the Body Composition Relationships

Total body weight (*BW*) is given by the combined mass of the fat (*FM*) and fat-free (*FFM*) compartments:

*BW* = *FM* + *FFM*

Taking the derivative with respect to time (days) gives:

$$\frac{\mathrm{dBW}}{\mathrm{dt}}=\frac{\mathrm{dFM}}{\mathrm{dt}}+\frac{\mathrm{dFFM}}{\mathrm{dt}}.$$

Application of the chain rule gives

$$\frac{\mathrm{dBW}}{\mathrm{dt}}=\frac{\mathrm{dFM}}{\mathrm{dt}}\;\;+\;\;\frac{\mathrm{dFFM}}{\mathrm{dFM}}\frac{\mathrm{dFM}}{\mathrm{dt}}=\left[\;1\;+\;\;\frac{\mathrm{dFFM}}{\mathrm{dFM}}\right]\frac{\mathrm{dFM}}{\mathrm{dt}}$$

which, after solving for *dFM*/*dt*, becomes:

$$\frac{\mathrm{dFM}}{\mathrm{dt}}=\left[\frac{1}{1\;+\;\;\mathrm{dFFM}/\mathrm{dFM}}\right]\frac{\mathrm{dBW}}{\mathrm{dt}}$$

Similarly,

$$\begin{array}{cc} \frac{\mathrm{dFFM}}{\mathrm{dt}} & =\frac{\mathrm{dBW}}{\mathrm{dt}}\;\;-\;\;\frac{\mathrm{dFM}}{\mathrm{dt}}\\ & =\frac{\mathrm{dBW}}{\mathrm{dt}}\;\;-\;\;\left[\frac{1}{1\;+\;\;\mathrm{dFFM}/\mathrm{dFM}}\right]\frac{\mathrm{dBW}}{\mathrm{dt}}\\ & =\left[1\;\;-\;\;\;\frac{1}{1\;+\;\;\mathrm{dFFM}/\mathrm{dFM}}\right]\frac{\mathrm{dBW}}{\mathrm{dt}}\\ & =\left[\frac{\mathrm{dFFM}/\mathrm{dFM}}{1\;+\;\;\mathrm{dFFM}/\mathrm{dFM}}\;\right]\frac{\mathrm{dBW}}{\mathrm{dt}} \end{array}$$

These relationships allow the temporal changes in body composition to be expressed as follows:

$$\begin{array}{cc} \frac{\mathrm{dFM}}{\mathrm{dt}} & =\left[\frac{1}{1\;\;+\;\;\mathrm{dFFM}/\mathrm{dFM}}\right]\frac{\mathrm{dBW}}{\mathrm{dt}},\\ \frac{\mathrm{dFFM}}{\mathrm{dt}} & =\left[\frac{\mathrm{dFFM}/\mathrm{dFM}}{1\;\;+\;\;\mathrm{dFFM}/\mathrm{dFM}}\right]\frac{\mathrm{dBW}}{\mathrm{dt}} \end{array}$$

### Appendix A.4. Derivation of Equation (3)

Inserting the body composition relationships into Equation (1) gives:

$$\begin{array}{cc} \mathrm{EI}-\mathrm{EE} & =\rho_{\mathrm{FM}}\frac{\mathrm{dFM}}{\mathrm{dt}}+\rho_{\mathrm{FFM}}\frac{\mathrm{dFFM}}{\mathrm{dt}}\\ & =\rho_{\mathrm{FM}}\left[\frac{1}{1+\mathrm{dFFM}/\mathrm{dFM}}\right]\frac{\mathrm{dBW}}{\mathrm{dt}}+\rho_{\mathrm{FFM}}\left[\frac{\mathrm{dFFM}/\mathrm{dFM}}{1\;+\mathrm{dFFM}/\mathrm{dFM}}\right]\frac{\mathrm{dBW}}{\mathrm{dt}}\\ & =\left[\frac{\;\rho_{\mathrm{FM}}+\rho_{\mathrm{FFM}}\mathrm{dFFM}/\mathrm{dFM}}{1\;+\mathrm{dFFM}/\mathrm{dFM}\;}\right]\frac{\mathrm{dBW}}{\mathrm{dt}} \end{array}$$

where the term enclosed in square brackets corresponds to the body mass energy density (*ρ_BM_*), defined as follows:

$$\rho_{\mathrm{BM}}=\frac{\rho_{\mathrm{FM}}+\rho_{\mathrm{FFM}}\mathrm{dFFM}/\mathrm{dFM}}{1+\mathrm{dFFM}/\mathrm{dFM}\;}$$

The preceding expression can therefore be written as follows:

$$\mathrm{EI}-\mathrm{EE}=\rho_{\mathrm{BM}}\frac{\mathrm{dBW}}{\mathrm{dt}}$$

Solving for *dBW*/*dt* gives Equation (3):

$$\frac{\mathrm{dBW}}{\mathrm{dt}}=\frac{\mathrm{EI}-\mathrm{EE}}{\rho_{\mathrm{BM}}}$$

### Appendix A.5. Invariance of the Solution to System (14) with Respect to the Choice of Unknown Variables

The independence of the solution to system (14) from the choice of unknown variables can be demonstrated by solving system (15) using an alternative set of unknowns. Consider, for example, the case in which *COx_net_* and *POx_net_* are selected as the unknown variables. Under this formulation, system (15) becomes:

$$\begin{array}{cc} \mathrm{CO}x_{\mathrm{net}}\;+\;\mathrm{PO}x_{\mathrm{net}} & =CI_{a}\;+PI_{a}-k_{\mathrm{eff}}N_{\mathrm{insensible}}\\ \rho_{C}\mathrm{CO}x_{\mathrm{net}}+\;\rho_{P}\;\mathrm{PO}x_{\mathrm{net}} & =\rho_{C}CI_{a}+\rho_{P}PI_{a}\; \end{array}$$

which can be expressed in matrix form as follows:

$$\left[\begin{array}{cc} 1 & 1\\ \rho_{C} & \rho_{P} \end{array}\right]\left[\begin{array}{c} \mathrm{CO}x_{\mathrm{net}}\;\\ \mathrm{PO}x_{\mathrm{net}} \end{array}\right]=\left\{\begin{array}{c} CI_{a}+\;PI_{a}-k_{\mathrm{eff}}N_{\mathrm{insensible}}\\ \rho_{C}CI_{a}\;+\rho_{P}PI_{a}\; \end{array}\right\}$$

Matrix inversion yields:

$$\begin{array}{cc} \left[\begin{array}{c} \mathrm{CO}x_{\mathrm{net}}\;\\ \mathrm{PO}x_{\mathrm{net}} \end{array}\right] & =\frac{1}{\rho_{P}-\rho_{C}}\left[\begin{array}{cc} \rho_{P} & -1\\ -\rho_{C} & 1 \end{array}\right]\left\{\begin{array}{c} CI_{a}+\;PI_{a}-k_{\mathrm{eff}}N_{\mathrm{insensible}}\\ \rho_{C}CI_{a}+\;\rho_{P}PI_{a}\; \end{array}\right\}\\ & =\left[\begin{array}{l} CI_{a}-\left(\frac{\rho_{P}}{\rho_{P}-\rho_{C}}\right)k_{\mathrm{eff}}N_{\mathrm{insensible}}\\ PI_{a}+\left(\frac{\rho_{C}}{\rho_{P}-\rho_{C}}\right)k_{\mathrm{eff}}N_{\mathrm{insensible}} \end{array}\right] \end{array}$$

Accordingly, the corresponding solution to (14) is:

$$\begin{array}{c} \mathrm{FO}x_{\mathrm{net}}\;=FI_{a},\\ \mathrm{CO}x_{\mathrm{net}}=CI_{a}-\left(\frac{\rho_{P}}{\rho_{P}-\rho_{C}}\right)k_{\mathrm{eff}}N_{\mathrm{insensible}},\\ \mathrm{PO}x_{\mathrm{net}}=PI_{a}+\left(\frac{\rho_{C}}{\rho_{P}-\rho_{C}}\right)k_{\mathrm{eff}}N_{\mathrm{insensible}}\; \end{array}$$

This result is identical to that obtained when the absorbed macronutrient fluxes were selected as the unknown variables. Thus, the solution of (14) does not depend on the particular choice of unknown quantities, completing the demonstration.

### Appendix A.6. Derivation of the Indirect Calorimetry Formula for Energy Expenditure and the Corresponding Rate of Metabolically Associated Material Elimination

For each gram of oxidized macronutrient, the corresponding oxygen consumption and carbon dioxide production are given by the following stoichiometric constants:

Carbohydrate: *a*_C_ L O_2_/g; *b*_C_ L CO_2_/g

Fat: *a*_F_ L O_2_/g; *b*_F_ L CO_2_/g

Protein: *a*_P_ L O_2_/g; *b*_P_ L CO_2_/g

Note that the stoichiometric constants are left in symbolic form to emphasize that the derivation and resulting equations arise from the structure of the oxidation relationships themselves rather than from any particular numerical values of the coefficients, which can be readily obtained from standard indirect calorimetry references [30,35,36,37].

Accordingly, the measured daily volumes of molecular oxygen consumed (VO_2_) and carbon dioxide produced (VCO_2_) satisfy the following relationships [30,35,36,37]:

$$\begin{array}{l} a_{C}\times \mathrm{CO}x_{\mathrm{net}}+a_{F}\times \mathrm{FO}x_{\mathrm{net}}+a_{P}\times \mathrm{PO}x_{\mathrm{net}}=VO_{2}\\ b_{C}\times \mathrm{CO}x_{\mathrm{net}}+b_{F}\times \mathrm{FO}x_{\mathrm{net}}+b_{P}\times \mathrm{PO}x_{\mathrm{net}}=\mathrm{VC}O_{2} \end{array}$$

In addition, net protein oxidation can be estimated from urinary nitrogen excretion because approximately 1 g of urinary nitrogen is generated for every *k_P_* g of oxidized protein:

*POx_net_* = *k_P_* × *N*_urine_

Therefore, the system can be rewritten as follows:

$$\begin{array}{l} a_{C}\times \mathrm{CO}x_{\mathrm{net}}+\;a_{F}\times \mathrm{FO}x_{\mathrm{net}}\;=VO_{2}-a_{P}\times k_{P}\times N_{\mathrm{urine}}\\ b_{C}\times \mathrm{CO}x_{\mathrm{net}}+b_{F}\times \mathrm{FO}x_{\mathrm{net}}=\mathrm{VC}O_{2}-b_{P}\times k_{P}\times N_{\mathrm{urine}} \end{array}$$

which, in matrix form, becomes:

$$\left[\begin{array}{cc} a_{C} & a_{F}\\ b_{C} & b_{F} \end{array}\right]\left[\begin{array}{c} \mathrm{CO}x_{\mathrm{net}}\\ \mathrm{FO}x_{\mathrm{net}} \end{array}\right]=\left[\begin{array}{c} VO_{2}-a_{P}\times k_{P}\times N_{\mathrm{urine}}\\ \mathrm{VC}O_{2}-b_{P}\times k_{P}\times N_{\mathrm{urine}} \end{array}\right]$$

Solving the system by matrix inversion yields:

$$\begin{array}{cc} \left[\begin{array}{c} \mathrm{CO}x_{\mathrm{net}}\\ \mathrm{FO}x_{\mathrm{net}} \end{array}\right] & =\frac{1}{a_{C}b_{F}-a_{F}b_{C}}\left[\begin{array}{cc} b_{F} & -a_{F}\\ -b_{C} & a_{C} \end{array}\right]\left[\begin{array}{c} VO_{2}-a_{P}\times k_{P}\times N_{\mathrm{urine}}\\ \mathrm{VC}O_{2}-b_{P}\times k_{P}\times N_{\mathrm{urine}} \end{array}\right]\\ & =\left[\begin{array}{c} \frac{b_{F}}{a_{C}b_{F}-a_{F}b_{C}}\times VO_{2}-\frac{a_{F}}{a_{C}b_{F}-a_{F}b_{C}}\times \mathrm{VC}O_{2}+\frac{\left(a_{F}b_{P}-a_{P}b_{F}\right)\times k_{P}}{a_{C}b_{F}-a_{F}b_{C}}\times N_{\mathrm{urine}}\\ -\frac{b_{C}}{a_{C}b_{F}-a_{F}b_{C}}\times VO_{2}+\frac{a_{C}}{a_{C}b_{F}-a_{F}b_{C}}\times \mathrm{VC}O_{2}+\frac{\left(a_{P}b_{C}-a_{C}b_{P}\right)\times k_{P}}{a_{C}b_{F}-a_{F}b_{C}}\times N_{\mathrm{urine}} \end{array}\right] \end{array}$$

The resulting expressions for net carbohydrate and fat oxidation are therefore:

$$\begin{array}{cc} \mathrm{CO}x_{\mathrm{net}} & =\frac{b_{F}}{a_{C}b_{F}-a_{F}b_{C}}\times VO_{2}\;-\frac{a_{F}}{a_{C}b_{F}-a_{F}b_{C}}\times \mathrm{VC}O_{2}+\frac{\left(a_{F}b_{P}-a_{P}b_{F}\right)\times k_{P}}{a_{C}b_{F}-a_{F}b_{C}}\times N_{\mathrm{urine}}\\ \mathrm{FO}x_{\mathrm{net}} & =-\frac{b_{C}}{a_{C}b_{F}-a_{F}b_{C}}\times VO_{2}+\;\frac{a_{C}}{a_{C}b_{F}-a_{F}b_{C}}\times \mathrm{VC}O_{2}+\frac{\left(a_{P}b_{C}-a_{C}b_{P}\right)\times k_{P}}{a_{C}b_{F}-a_{F}b_{C}}\times N_{\mathrm{urine}} \end{array}$$

Energy expenditure (*EE*) is then given by:

$$\begin{array}{cc} \mathrm{EE} & =\rho_{C}\mathrm{CO}x_{\mathrm{net}}+\rho_{F}\mathrm{FO}x_{\mathrm{net}}+\rho_{P}\mathrm{PO}x_{\mathrm{net}}\\ & =\frac{\rho_{C}b_{F}-\rho_{F}b_{C}}{a_{C}b_{F}-a_{F}b_{C}}\times VO_{2}\;+\;\frac{\rho_{F}a_{C}-\rho_{C}a_{F}}{a_{C}b_{F}-a_{F}b_{C}}\times \mathrm{VC}O_{2}+\;\left(\frac{\rho_{C}\left(a_{F}b_{P}-a_{P}b_{F}\right)+\rho_{F}\left(a_{P}b_{C}-a_{C}b_{P}\right)}{a_{C}b_{F}-a_{F}b_{C}}+\rho_{P}\right)\times k_{P}\times N_{\mathrm{urine}}\\ & =e_{1}\times VO_{2}+\;e_{2}\times \mathrm{VC}O_{2}+e_{3}\times N_{\mathrm{urine}} \end{array}$$

where

$$\begin{array}{c} e_{1}=\frac{\rho_{C}b_{F}-\rho_{F}b_{C}}{a_{C}b_{F}-a_{F}b_{C}},\\ e_{2}=\frac{\rho_{F}a_{C}-\rho_{C}a_{F}}{a_{C}b_{F}-a_{F}b_{C}},\\ e_{3}=\left(\frac{\rho_{C}\left(a_{F}b_{P}-a_{P}b_{F}\right)+\rho_{F}\left(a_{P}b_{C}-a_{C}b_{P}\right)}{a_{C}b_{F}-a_{F}b_{C}}+\rho_{P}\right)\times k_{P} \end{array}$$

The corresponding rate of metabolically associated material elimination (*M_EE_*) is given by:

$$\begin{array}{cc} M_{\mathrm{EE}} & =\mathrm{CO}x_{\mathrm{net}}+\mathrm{FO}x_{\mathrm{net}}+\mathrm{PO}x_{\mathrm{net}}\\ & =\frac{b_{F}-b_{C}}{a_{C}b_{F}-a_{F}b_{C}}\times VO_{2}\;+\;\frac{a_{C}-a_{F}}{a_{C}b_{F}-a_{F}b_{C}}\times \mathrm{VC}O_{2}+\;\left(\frac{a_{F}b_{P}-a_{P}b_{F}+\;a_{P}b_{C}-a_{C}b_{P}}{a_{C}b_{F}-a_{F}b_{C}}+1\right)\times k_{P}\times N_{\mathrm{urine}}\\ & =m_{1}\times VO_{2}+\;m_{2}\times \mathrm{VC}O_{2}+m_{3}\times N_{\mathrm{urine}} \end{array}$$

where

$$\begin{array}{c} m_{1}=\frac{b_{F}-b_{C}}{a_{C}b_{F}-a_{F}b_{C}},\\ m_{2}=\frac{a_{C}-a_{F}}{a_{C}b_{F}-a_{F}b_{C}},\\ m_{3}=\left(\frac{a_{F}b_{P}-a_{P}b_{F}+\;a_{P}b_{C}-a_{C}b_{P}}{a_{C}b_{F}-a_{F}b_{C}}+1\right)\times k_{P} \end{array}$$

Therfore, the general mathematical forms of *EE* and *M_EE_* are:

$$\begin{array}{cc} \mathrm{EE} & =e_{1}\times VO_{2}+\;e_{2}\times \mathrm{VC}O_{2}+e_{3}\times N_{\mathrm{urine}}\\ M_{\mathrm{EE}} & =m_{1}\times VO_{2}+\;m_{2}\times \mathrm{VC}O_{2}+m_{3}\times N_{\mathrm{urine}} \end{array}$$

where the coefficient vectors (*e*_1_, *e*_2_, *e*_3_) and (*m*_1_, *m*_2_, *m*_3_) are not proportional. Consequently, *M_EE_* is not proportional to *EE*. Thus, although both quantities are derived from the same underlying substrate-oxidation processes, the same energy expenditure need not correspond to the same rate of metabolically associated material elimination.

## References

[1] Antonetti V.W. (1973). The equations governing bodyweight change in human beings. Am. J. Clin. Nutr..

[2] Alpert S.S. (1979). A two-reservoir energy model of the human body. Am. J. Clin. Nutr..

[3] Alpert S.S. (1990). Growth, thermogenesis, and hyperphagia. Am. J. Clin. Nutr..

[4] Alpert S.S. (2005). A limit on the energy transfer rate from the human fat store in hypophagia. J. Theor. Biol..

[5] Weinsier R.L., Bracco D., Schutz Y. (1993). Predicted effects of small decreases in energy expenditure on weight gain in adult women. Int. J. Obes. Relat. Metab. Disord..

[6] Westerterp K.R., Donkers J.H., Fredrix E.W., Boekhoudt P. (1995). Energy intake, physical activity and body weight: A simulation model. Br. J. Nutr..

[7] Christiansen E., Garby L. (2002). Prediction of body weight changes caused by changes in energy balance. Eur. J. Clin. Investig..

[8] Flatt J.P. (2004). Carbohydrate-fat interactions and obesity examined by a two-compartment computer model. Obes. Res..

[9] Christiansen E., Garby L., Sørensen T.I. (2005). Quantitative analysis of the energy requirements for development of obesity. J. Theor. Biol..

[10] Song B., Thomas D.M. (2007). Dynamics of starvation in humans. J. Math. Biol..

[11] Chow C.C., Hall K.D. (2008). The dynamics of human body weight change. PLoS Comput. Biol..

[12] Hall K.D. (2010). Predicting metabolic adaptation, body weight change, and energy intake in humans. Am. J. Physiol. Endocrinol. Metab..

[13] Thomas D.M., Scioletti M., Heymsfield S.B. (2019). Predictive Mathematical Models of Weight Loss. Curr. Diab. Rep..

[14] Hall K.D. (2006). Computational model of in vivo human energy metabolism during semistarvation and refeeding. Am. J. Physiol. Endocrinol. Metab..

[15] Hall K.D., Jordan P.N. (2008). Modeling weight-loss maintenance to help prevent body weight regain. Am. J. Clin. Nutr..

[16] Kozusko F.P. (2001). Body weight setpoint, metabolic adaption and human starvation. Bull. Math. Biol..

[17] Kozusko F.P. (2002). The effects of body composition on setpoint-based weight loss. Math. Comput. Model..

[18] Kondepudi D., Prigogine I. (1998). Modern Thermodynamics: From Heat Engines to Dissipative Structures.

[19] Bejan A. (2016). Advanced Engineering Thermodynamics.

[20] Tomé D., Bos C. (2000). Dietary protein and nitrogen utilization. J. Nutr..

[21] Dickerson R.N. (2016). Nitrogen Balance and Protein Requirements for Critically Ill Older Patients. Nutrients.

[22] Rand W.M., Pellett P.L., Young V.R. (2003). Meta-analysis of nitrogen balance studies for estimating protein requirements in healthy adults. Am. J. Clin. Nutr..

[23] Suzuki D., Hayamizu K., Uno C., Hasegawa Y., Kuwahata M., Kido Y., Suzuki Y. (2025). Nitrogen Requirements in Healthy Adults: A Systematic Review and Meta-Analysis of Nitrogen Balance Studies. Nutrients.

[24] Arencibia-Albite F. (2026). A numerical and analytical evaluation of the consistency of the energy balance model identifies limitations and systematic relationships between mass intake and body weight dynamics. Front. Nutr..

[25] Arencibia-Albite F. (2022). The energy balance theory is an inconsistent paradigm. J. Theor. Biol..

[26] Hall K.D., Heymsfield S.B., Kemnitz J.W., Klein S., Schoeller D.A., Speakman J.R. (2012). Energy balance and its components: Implications for body weight regulation. Am. J. Clin. Nutr..

[27] Hall K.D., Ayuketah A., Brychta R., Cai H., Cassimatis T., Chen K.Y., Chung S.T., Costa E., Courville A., Darcey V. (2019). Ultra-Processed Diets Cause Excess Calorie Intake and Weight Gain: An Inpatient Randomized Controlled Trial of Ad Libitum Food Intake. Cell Metab..

[28] Thomas D.M., Bouchard C., Church T., Slentz C., Kraus W.E., Redman L.M., Martin C.K., Silva A.M., Vossen M., Westerterp K. (2012). Why do individuals not lose more weight from an exercise intervention at a defined dose? An energy balance analysis. Obes. Rev..

[29] Hall K.D., Bemis T., Brychta R., Chen K.Y., Courville A., Crayner E.J., Goodwin S., Guo J., Howard L., Knuth N.D. (2015). Calorie for Calorie, Dietary Fat Restriction Results in More Body Fat Loss than Carbohydrate Restriction in People with Obesity. Cell Metab..

[30] Frayn K.N. (1983). Calculation of substrate oxidation rates in vivo from gaseous exchange. J. Appl. Physiol. Respir. Environ. Exerc. Physiol..

[31] Hall K.D. (2008). What is the required energy deficit per unit weight loss?. Int. J. Obes..

[32] Forbes G.B. (1987). Lean body mass-body fat interrelationships in humans. Nutr. Rev..

[33] Alhotan R.A., Pesti G.M., Billard L. (2024). The linear relationship between true protein and nitrogen contents of feed and food ingredients: Calculating true protein from a new conversion factor. Cogent Food Agric..

[34] Mariotti F., Tomé D., Mirand P.P. (2008). Converting nitrogen into protein-beyond 6.25 and Jones’ factors. Crit. Rev. Food Sci. Nutr..

[35] Ferrannini E. (1988). The theoretical bases of indirect calorimetry: A review. Metabolism.

[36] Weir J.B. (1949). New methods for calculating metabolic rate with special reference to protein metabolism. J. Physiol..

[37] Hall K.D., McCue M.D. (2012). Quantitative Physiology of Human Starvation: Adaptations of Energy Expenditure, Macronutrient Metabolism and Body Composition. Comparative Physiology of Fasting, Starvation, and Food Limitation.

[38] Livesey G., Elia M. (1988). Estimation of energy expenditure, net carbohydrate utilization, and net fat oxidation and synthesis by indirect calorimetry: Evaluation of errors with special reference to the detailed composition of fuels. Am. J. Clin. Nutr..

[39] Hall K.D., Farooqi S., Friedman J.M., Klein S., Loos R.J.F., Mangelsdorf D.J., O’Rahilly S., Ravussin E., Redman L.M., Ryan D.H. (2022). The energy balance model of obesity: Beyond calories in, calories out. Am. J. Clin. Nutr..

[40] Ludwig D.S., Aronne L.J., Astrup A., de Cabo R., Cantley L.C., Friedman M.I., Heymsfield S.B., Johnson J.D., King J.C., Krauss R.M. (2021). The Carbohydrate-Insulin Model: A Physiological Perspective on the Obesity Pandemic. Am. J. Clin. Nutr..

[41] Hall K.D., Guo J., Speakman J.R. (2019). Do low-carbohydrate diets increase energy expenditure?. Int. J. Obes..

[42] Arencibia-Albite F. (2020). Serious analytical inconsistencies challenge the validity of the energy balance theory. Heliyon.

[43] Velleman D.J. (2019). How to Prove It: A Structured Approach.

[44] Chartrand G., Polimeni A.D., Zhang P. (2018). Mathematical Proofs: A Transition to Advanced Mathematics.

[45] Subar A.F., Kipnis V., Troiano R.P., Midthune D., Schoeller D.A., Bingham S., Sharbaugh C.O., Trabulsi J., Runswick S., Ballard-Barbash R. (2003). Using intake biomarkers to evaluate the extent of dietary misreporting in a large sample of adults: The OPEN study. Am. J. Epidemiol..

[46] Mahabir S., Baer D.J., Giffen C., Subar A., Campbell W., Hartman T.J., Clevidence B., Albanes D., Taylor P.R. (2006). Calorie intake misreporting by diet record and food frequency questionnaire compared to doubly labeled water among postmenopausal women. Eur. J. Clin. Nutr..

[47] Winkler J.T. (2005). The fundamental flaw in obesity research. Obes. Rev..

[48] Dhurandhar N.V., Schoeller D., Brown A.W., Heymsfield S.B., Thomas D., Sørensen T.I., Speakman J.R., Jeansonne M., Allison D.B., Energy Balance Measurement Working Group (2015). Energy balance measurement: When something is not better than nothing. Int. J. Obes..

[49] Toth M.J., Sites C.K., Poehlman E.T. (1999). Hormonal and physiological correlates of energy expenditure and substrate oxidation in middle-aged, premenopausal women. J. Clin. Endocrinol. Metab..

[50] Egan A.M., Collins A.L. (2022). Dynamic changes in energy expenditure in response to underfeeding: A review. Proc. Nutr. Soc..

[51] Day D.S., Gozansky W.S., Van Pelt R.E., Schwartz R.S., Kohrt W.M. (2005). Sex hormone suppression reduces resting energy expenditure and β-adrenergic support of resting energy expenditure. J. Clin. Endocrinol. Metab..

[52] Tsigos C., Kyrou I., Kassi E., Chrousos G.P., Feingold K.R., Ahmed S.F. (2000). Stress: Endocrine Physiology and Pathophysiology. Endotext.

[53] Hopkins M., Beaulieu K., Gibbons C., Halford J.C.G., Blundell J., Stubbs J., Finlayson G., Feingold K.R. (2000). The Control of Food Intake in Humans. Endotext.

[54] McCrickerd K., Forde C.G. (2016). Sensory influences on food intake control: Moving beyond palatability. Obes. Rev..

[55] Forde C.G., de Graaf K. (2022). Influence of Sensory Properties in Moderating Eating Behaviors and Food Intake. Front. Nutr..

[56] Finlayson G., Dalton M. (2012). Hedonics of food consumption: Are food “liking” and “wanting” viable targets for appetite control in the obese?. Curr. Obes. Rep..

